# Assessing the clinical and cost-effectiveness of endovascular vs open revascularisation in severe occlusive aorto-iliac disease (EVOCC trial): study protocol for a randomised controlled trial

**DOI:** 10.1136/bmjopen-2025-106474

**Published:** 2025-10-07

**Authors:** Athanasios Saratzis, Alun Davies, Athanasios Diamantopoulos, Robert S M Davies, David Epstein, Marcus Jepson, Daniel Perez, Despina Apergi, Keith Jonathan Harris, Hany Zayed, Cassandra Brookes, Shaun Barber, Ana Suazo Di Paola, Luke Ingram, Carla Richardson, Anish Patel

**Affiliations:** 1 Cardiovascular Sciences, University of Leicester Medical School, Leicester, UK; 2 University of Leicester, Leicester, UK; 3 Vascular, Imperial College London, London, UK; 4 Interventional Radiology, Guy's and St Thomas’ Hospitals NHS Trust, London, UK; 5 Leicester Vascular Institute, University Hospitals of Leicester NHS Trust, Leicester, UK; 6 Faculty of Economic and Business Sciences, University of Granada, Granada, Spain; 7 University of Bristol School of Social and Community Medicine, Bristol, UK; 8 Vascular Surgery, Guy's and St Thomas’ NHS Foundation Trust Cardiovascular Centre, London, UK; 9 Leicester Clinical Trials Unit, University of Leicester, Leicester, UK

**Keywords:** Cardiovascular imaging, Cardiovascular Disease, Interventional radiology, SURGERY, Vascular surgery

## Abstract

**Introduction:**

Severe aorto-iliac steno-occlusive atherosclerotic disease is a major cause of morbidity and amputation in patients with peripheral arterial disease. While both open surgical and endovascular revascularisation are standard treatments in this patient group, there is no high-quality randomised evidence to determine which approach offers superior clinical and cost-effectiveness, leading to uncertainty and poor outcomes after intervention.

**Methods and analysis:**

The EVOCC trial is a national, multicentre, parallel-group, superiority randomised controlled trial comparing open surgery to endovascular revascularisation in patients with symptomatic severe aorto-iliac occlusive disease. A total of 628 participants across 30 NHS sites in the UK will be randomised 1:1 to receive either open surgery or endovascular (minimally invasive) intervention. The primary outcome is amputation-free survival, defined as time to first event (major lower limb amputation or death). Secondary outcomes include mortality, cardiovascular events, hospital readmissions, re-interventions and quality-of-life measures. An internal pilot phase (10 sites, 6-month duration) will assess recruitment feasibility. A QuinteT Recruitment Intervention is integrated into the trial to optimise recruitment.

**Ethics and dissemination:**

The trial has received ethical approval from a UK Research Ethics Committee (REC reference: 23/SW/0065; trial registration reference: ISRCTN14591444). Informed consent will be obtained from all participants.

The EVOCC trial is the first RCT assessing the clinical and cost-effectiveness of open vs endovascular revascularisation for severe aorto-iliac disease worldwide. The results will provide robust evidence to inform clinical practice and healthcare policies globally. Results will be disseminated via patient groups, online lay summaries, a trial website, social media, presentations in conferences, a formal scientific publication in a medical journal and direct communications with policymakers across borders.

**Trial registration number:**

ISRCTN14591444.

**Strengths and limitations of this study**

- Randomised controlled trial assessing clinical and cost-effectiveness of open vs endovascular surgery in people with aorto-iliac steno-occlusive disease, within secondary care.
- Pragmatic trial design with broad inclusion criteria, allowing translation to clinical care—all types of open and endovascular surgery allowed in the trial.
- Both inflow and outflow procedures are allowed to be performed as per local clinicians’ choice.
- Uniform follow-up for a minimum of 2 years per participant.
- Lay dissemination of findings and study progress embedded in the delivery of the trial.

## Introduction

### Background and rationale

 Peripheral arterial disease (PAD) secondary to atherosclerosis is a significant cause of disability and the leading cause of lower limb amputations in most healthcare systems]. It affects more than 20% of people above age 55 in the UK and similar societies]. Interventions to revascularise the lower limbs of people with PAD are the most common arterial procedures in the National Health Service (NHS) and most countries worldwide]. Despite advances in surgical and endovascular (minimally invasive) PAD treatments, PAD remains a leading cause of mortality, morbidity and main reason for leg amputations.

Severe aorto-iliac occlusive disease is one of the most common anatomical variants in people who present with symptomatic PAD, for example, intermittent claudication (pain when walking) or chronic limb-threatening ischaemia (leg pain at rest or leg tissue loss)]. The Trans-Atlantic Inter-Society Consensus (TASC) guidelines] classify severe cases of aorto-iliac PAD as type C or D based on anatomical details such as the length and number of occlusions seen in the aorta or iliac arteries of these patients]. There is clinical equipoise regarding the best revascularisation strategy (open surgery vs endovascular treatment) in this group of patients (TASC C and D aorto-iliac PAD) since the introduction of modern endovascular technologies into routine PAD care]. While open surgery is associated with lower long-term re-intervention rates, it carries higher perioperative risks. Endovascular interventions are less invasive but have high long-term failure rates. Observational data suggest a survival benefit with open surgery, but robust randomised controlled trial (RCT) based evidence is non-existent].

### Objectives

- **Primary objective:** to determine whether open surgery offers superior clinical and cost-effectiveness compared to endovascular revascularisation in severe aorto-iliac disease (TASC II C/D classification).
- **Secondary objectives:** to evaluate amputation-free survival, mortality, cardiovascular events, hospital readmissions, re-interventions and cost-effectiveness.

## Methods and trial design

A multicentre, national, prospective, parallel-group RCT with an internal pilot phase will be performed, across the National Health Service (NHS).

### Methods: participants, interventions and outcomes

#### Study setting

The trial will be conducted at 30 NHS hospital sites across the UK, including both academic and non-academic centres providing specialist vascular care.

### Eligibility criteria

#### Inclusion criteria

- Adults (≥18 years) with symptomatic PAD (rest pain, tissue loss or lifestyle-limiting claudication).
- Severe aorto-iliac occlusive disease (TASC II C/D).
- Multidisciplinary team (MDT) consensus that both surgical and endovascular options are viable.
- Ability to provide informed consent.
- Ability to understand written English or availability of a translator to explain the trial documentation.

#### Exclusion criteria

- Asymptomatic PAD.
- Non-consent.
- Comorbid conditions precluding intervention.

### Interventions

#### Open surgery

Procedures may include aorto-(bi/uni)-femoral/iliac bypass, aortic or iliac endarterectomy, or other hybrid procedures.

#### Endovascular treatment

Angioplasty, stenting, vessel preparation strategies or adjunct endovascular procedures.

### Outcomes

#### Primary outcome

Amputation-free survival (time to death or major lower limb amputation) measured in days from randomisation until the first event or until the end of the trial (minimum 2 years).

#### Secondary outcomes

1. Mortality (all-cause), measured in days from randomisation until the first event or until the end of the trial (minimum 2 years) where no event occurs.
2. Cardiovascular events (myocardial infarction, stroke, admission for heart failure), measured in days from randomisation until the first event or until the end of the trial (minimum 2 years) where no event occurs.
3. Number of hospital admissions (including reasons for readmissions), following discharge from index surgical admission for up to 4.5 years at 6 and 12 months and annually thereafter.
4. All lower limb amputations (minor and major, that is, below and above ankle joint) measured in days from randomisation until the first event or until the end of the trial (minimum 2 years) where no event occurs at baseline, trial intervention visit and day of discharge visit and all follow-up visits (30 days, 6 and 12 months and annually thereafter).
5. Concomitant medications (specifically: use of statin, use of antithrombotic medication) at the day of discharge, 30 days, 6 months, 12 months and annually thereafter.
6. Estimated glomerular filtration rate (renal function) based on serum creatinine levels using the chronic kidney disease Epidemiology formula measured at 6 and 12 months and annually thereafter.
7. Low-density lipoprotein measured using standard blood sample assessments (standard of care, not research) at baseline and 6 months follow-up visit.
8. HbA1c measured using standard blood sample assessments (standard of care, not research) at baseline and 6 months follow-up visit.
9. Cholesterol was measured using standard blood sample assessments (standard of care, not research) at baseline and 6 months follow-up visit.
10. Ankle brachial pressure index measured using a sphygmomanometer and a hand-held Doppler device at baseline, 6 and 12 months and annually up to 54 months.
11. Types of endovascular devices used and/or name of surgery performed (open) recorded at trial intervention visit and day of discharge.
12. Quality of life measured using the EQ-5D-5L measured at 6 and 12 months and annually thereafter.
13. Healthcare resource use and costs measured using economic evaluation led by Health Economists with considerable experience in National Institute for Healthcare Research Health Technology Assessment (NIHR HTA)-funded vascular research across follow-up assessments over a minimum of 24 months and until the end of the trial.

### Statistical considerations

#### Sample size calculation

Based on a 20.63% event rate per year for patients allocated to endovascular treatment in terms of amputation and/or death (national Hospital Episodes Statistics (HES) data), the proportion of patients for this arm free from death and amputation at 3 years is expected to be 50%. Calculations are based on our published meta-analysis, cohort study^1 2^ and NHS event rates in an HES analysis for this trial.^3^ Assuming a recruitment period of 2 years and a median follow-up time of 3 years, a sample size of 596 patients (accruing 256 events during follow-up) will provide 90% power (two sided, a=0.05) to detect an absolute risk reduction of 13.3% (HR of 0.66, 50% vs 63.3%) for the primary outcome at 3 years in patients having open surgery vs endovascular treatment. This is equivalent to an increase in restricted mean survival time (death and/or amputation) of 3 months between arms. Allowing for 5% loss to follow-up data, the final sample size is 628. Patients will be minimised based on age, sex and presence of tissue loss and will be stratified by site. An exploratory subgroup analysis will be performed based on the extent of aortic or iliac artery occlusions and configuration of endovascular treatment.

#### Randomisation

Randomisation (1:1) will be performed via a web-based system (Sealed Envelope) stratified by site and minimised by age, sex and presence of tissue loss.

#### Blinding

Blinding is not feasible due to the nature of interventions, but outcome assessors will be blinded.

### Data analysis

The Leicester Clinical Trials Unit (LCTU) statistician will be responsible for planning, monitoring and carrying out data analyses throughout the study. Statistical analysis will be undertaken according to a pre-specified statistical analysis plan written prior to database lock and reported following CONSORT statements].

The primary outcome (time to first event, death or major lower limb amputation) will be calculated by measuring the time (in days) from randomisation until the first event or last known event-free observation during the follow-up period. Patients not experiencing a primary outcome event will have their time to event censored at the last follow-up date. The primary analysis of the primary outcome will be undertaken on an intention-to-treat basis. Secondary analysis of the primary outcome will use a per-protocol population excluding major protocol deviators. The primary outcome will be compared between randomised arms using restricted mean survival time carried out using a validated programme such as the Stata module ‘STRMST2’ or equivalent. A point estimate, 95% CI and two-sided p value will be presented. Statistical significance will occur if the p value is <0.05. The primary analysis will be adjusted for the minimisation and stratification factors used at randomisation.

Unpowered exploratory subgroup analyses of the primary outcome will be performed to assess associations between TASC class of aorto-iliac disease, length and location of aortic and iliac occlusions, and types of endovascular treatment (eg, use of kissing stents, aorto-iliac stents, use of covered vs uncovered stents and use vs no use of an aortic stent).

Secondary outcomes will be analysed on a complete case population and reported similarly to the primary outcome using appropriate methodology to compare randomised groups; time to event using restricted mean survival analysis or cox proportional hazards regression (as appropriate), binary outcomes using logistic regression, continuous outcomes using linear regression or where not normally distributed using appropriate non-parametric methods.

### Measurement of costs and outcomes

A prospectively planned economic evaluation will be conducted from an NHS and personal social services perspective,^3 4^ as per National Institute of Care Excellence (NICE) recommendations], led by Health Economists with considerable experience in NIHR HTA-funded vascular research. The use of healthcare services during the index admission, procedure, major clinical events, re-interventions, accident and emergency, outpatients and primary care will be recorded over the trial time horizon through the Case Record Forms. The quality of life will be assessed using the EQ-5D-5L, converted to health utility scores using the UK value set recommended by NICE. Unit costs will be based on manufacturers’ prices and national databases. Days lost from work and activities will be recorded and used in a secondary analysis. The extent and pattern of missing data will be assessed and appropriate methods employed, for example, multiple imputation.

If the trial finds a clinically meaningful difference in outcomes or costs over the study time horizon, a decision model will be constructed to take account of the loss in life years and long-term impact on quality of life after complications. A state-transition decision model (Markov model) will estimate costs and quality-adjusted life years over the lifetime of the cohort. The recommended discount rate of 3.5% per year will be used. Parametric survival analysis will be used to estimate rates of death, cardiovascular events and amputations. Long-term rates of events will be estimated based on extrapolation of study data and literature. Outcomes or states of the model will be decided based on consultation with experts and review of previous economic evaluations in PAD. Tunnel states may be used if mortality rates following non-fatal major events change over time. Other cause mortality rates by age and sex will be included from national life tables, calibrated for the PAD population. Sensitivity analyses will be conducted by varying key variables with high uncertainty. The model will be constructed, analysed and reported according to international guidelines].

### Data management and monitoring

- Data will be collected via appropriate approved electronic case report forms and software (secure and encrypted) and managed by LCTU.
- An independent Data Monitoring Committee will oversee the trial.
- Serious adverse events will be reported per NHS established Good Clinical Practice guidelines.

## Ethics and dissemination

### Ethical approval and consent

The trial has received ethical approval from the UK Research Ethics Committee (REC reference: 23/SW/0065). Informed consent will be obtained from all participants. A consent form is included as an appendix (online supplemental appendix 1).

### Dissemination plan

Results will be published in peer-reviewed journals, presented at vascular conferences and shared with patient groups and policymakers.

### Trial status

Protocol Version: 2.1 (27 February 2025).

Recruitment start date: 01 October 2023.

Planned recruitment end date: 30 September 2028.

The internal pilot was successfully completed in April 2024, and the trial has progressed to the main phase of recruitment, as per the original plan.

### Patient and public involvement (PPI) during study delivery

PPI plays a crucial role in study delivery, ensuring that the study remains patient-centred and addresses the priorities of those affected by symptomatic PAD which requires revascularisation. From the early design stages, two dedicated PPI co-applicants and an advisory group have actively shaped the trial’s methodology, ensuring that recruitment, consent, follow-up and dissemination strategies align with patient needs and experiences.

One of the key contributions of PPI has been in refining the primary outcome measures to reflect what matters most to patients—amputation-free survival, including both death and major lower limb amputations. Patients also highlighted concerns over the high rate of re-interventions following endovascular treatment, which reinforced the need for long-term follow-up to assess treatment durability and quality of life.

To facilitate recruitment, the study has adopted tailored consent procedures, particularly for frail patients and those with cognitive impairment. PPI representatives have helped develop patient-friendly materials, ensuring clarity and accessibility. The inclusion of translated documents and targeted outreach to ethnically and socio-economically diverse populations further enhances inclusivity.

Throughout the trial, a dedicated PPI advisory group will provide continuous oversight, meeting quarterly to review trial progress, ensure participant support and advise on potential challenges. This group will work closely with the Trial Management Group to address any concerns raised by participants and improve engagement.

In terms of dissemination, PPI co-applicants will lead the preparation of regular lay summaries, shared via social media, patient advocacy groups and national vascular societies. By embedding patient and public voices at every stage, EVOCC ensures that the study remains clinically relevant, ethically robust and accessible, ultimately improving care and decision-making for patients with severe aorto-iliac occlusive disease.

#### QuinteT recruitment intervention (QRI)

A QRI has been embedded in the study. QRI-informed training will be applied as part of the study site set-up meetings. Thereafter, a range of methods of data collection and analysis will be applied so that sources of recruitment difficulties can be identified, and suggestions will be made to optimise recruitment.

## Discussion

The EVOCC trial is set to be an important study with the potential to shape the treatment of severe aorto-iliac occlusive disease for decades to come, not only in the NHS, but worldwide, given the paucity of effectiveness-driven studies in this area. It will compare the clinical and cost-effectiveness of open surgery vs endovascular treatment in patients with severe steno-occlusive PAD, a condition that leads to severe pain, ulceration, gangrene and a high risk of amputation or death. Despite both treatments being widely used, there is no randomised evidence to determine which approach offers superior outcomes. The trial will finally end this uncertainty, but it also comes with significant practical and operational challenges.

Recruitment and retention of participants pose a major challenge. Many patients with PAD are frail, have multiple long-term conditions or struggle with cognitive impairment, which makes consenting difficult. Given that the trial must recruit 628 patients across 30 NHS hospitals, a structured approach involving robust PPI, tailored consent processes and multidisciplinary support is needed. Equally challenging is ensuring equipoise among clinicians. While a national survey confirmed that 91% of vascular specialists believe in the need for this trial, local preferences and prior experience with either open or endovascular techniques may still influence recruitment. Maintaining engagement among surgeons, interventional radiologists and trial sites will be essential.

Another key challenge is the variability in treatment approaches. Open surgery and endovascular techniques require highly individualised planning, making it difficult to create a one-size-fits-all protocol.^4^ To ensure real-world relevance, the trial allows local MDTs to determine the exact procedure for each patient. Long-term follow-up is another operational hurdle, particularly as 43% of endovascular patients require re-interventions within 3 years. Structured assessments at 6 months, 1 year and annually thereafter, combined with data linkage to HES and Office for National Statistics records, will be vital in tracking long-term survival, amputation rates and healthcare costs in the long term (future separate study).

The economic implications of the trial are significant. Open surgery is associated with longer hospital stays and higher perioperative risks, whereas endovascular treatment involves expensive devices and frequent re-interventions]. A detailed cost-effectiveness analysis using a Markov model will guide NHS policy on whether the initial expense of endovascular treatment is justified by its long-term outcomes.

The impact of EVOCC will be significant across healthcare systems, shaping NHS care, international guidelines and clinical decision-making worldwide. If the trial confirms the superiority of open surgery, NHS resources may be reallocated to reduce reliance on costly endovascular interventions.^5^ Conversely, if endovascular treatment proves non-inferior, it may encourage a shift towards minimally invasive techniques, reducing hospital stays and perioperative complications. By providing definitive evidence, EVOCC will guide treatment strategies, resolving uncertainty and ensuring optimal care for patients.

## Supplementary material

10.1136/bmjopen-2025-106474 online supplemental appendix 1

10.1136/bmjopen-2025-106474 online supplemental appendix 2

## References

[1] Butcher NJ, Monsour A, Mew EJ (2022). Guidelines for Reporting Outcomes in Trial Reports: The CONSORT-Outcomes 2022 Extension. JAMA.

[2] (2013). Guide to the methods of technology appraisal 2013. NICE process and methods guides.

[3] Husereau D, Drummond M, Augustovski F (2022). Consolidated Health Economic Evaluation Reporting Standards 2022 (CHEERS 2022) Statement: Updated Reporting Guidance for Health Economic Evaluations. Value Health.

[4] Saratzis A, Argyriou A, Davies R (2022). Editor’s Choice - Covered vs. Bare Metal Stents in the Reconstruction of the Aortic Bifurcation: Early and Midterm Outcomes from the COBRA European Multicentre Registry. Eur J Vasc Endovasc Surg.

[5] Saratzis A, Torsello GB, Cardona-Gloria Y (2024). Cost Analysis of Target Lesion Revascularisation in Patients With Femoropopliteal In Stent Re-Stenosis or Occlusion: The COSTLY-TLR Study. Eur J Vasc Endovasc Surg.

