# Supplementary material for: Assessing the clinical and cost-effectiveness of endovascular vs open revascularisation in severe occlusive aorto-iliac disease (EVOCC trial): study protocol for a randomised controlled trial
Source: BMJ Open. 2025 Oct 7;15(10):e106474. doi: 10.1136/bmjopen-2025-106474 (PMC12506155; doi:10.1136/bmjopen-2025-106474)

**
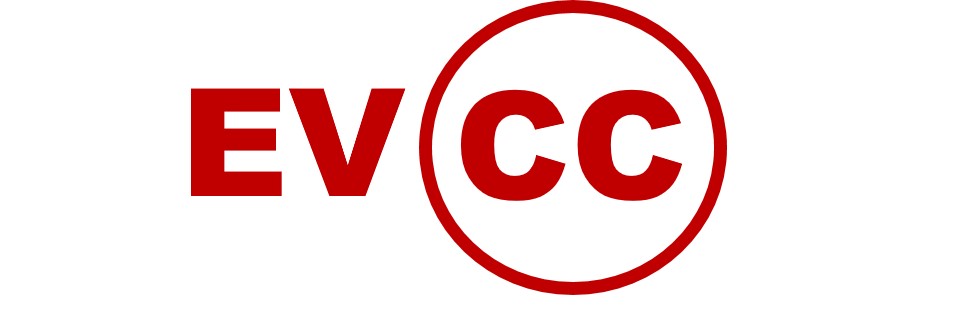
** **A randomised controlled trial assessing the clinical and cost-effectiveness of Endovascular vs. Open revascularisation in severe oCClusive aorto-iliac disease. The EVOCC Trial.**

| **SPONSOR** | University of Leicester (Ref: 0914) |
| --- | --- |
| **IRAS ID** | 323392 |
| **REC Reference Number** | 23/SW/0065 |
| **ISRCTN Reference** | ISRCTN14591444 |
| **FUNDER** | HTA Programme, NIHR151230 |

**Version 3.0;30/07/2025**


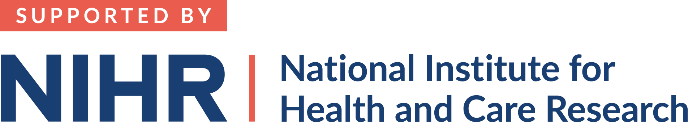


This project is funded by the NIHR [Health Technology Assessment Programme (HTA), Reference: NIHR151230]. The views ex pressed are those of the author(s) and not necessarily those of the NIHR or the Department of Health and Social Care.

**SIGNATURE PAGE**

The undersigned confirm that this protocol has been agreed and accepted and that the Chief Investigator agrees to conduct the trial in compliance with the approved protocol and will adhere to the principles outlined in the Medicines for Human Use (Clinical Trials) Regulations 2004 (SI 2004/1031), amended regulations (SI 2006/1928) and any subsequent amendments of the clinical trial regulations, GCP guidelines, the Sponsor’s (and any other relevant) SOPs, and other regulatory requirements.

I agree to ensure that the confidential information contained in this document will not be used for any other purpose other than the evaluation or conduct of the clinical investigation without the prior written consent of the Sponsor

I also confirm that I will make the findings of the trial publicly available through publication or other dissemination tools without any unnecessary delay and that an honest accurate and transparent account of the trial will be given. Any discrepancies and serious breaches of GCP from the trial as planned in this protocol will be explained.

| **Sponsor: University of Leicester** | |
| --- | --- |
| **Signature:** | Date: |
| **Name (please print):** |  |
| **Position:** |  |
| **Chief Investigator: Mr Athanasios Saratzis** | |
| Signature: | Date: |
| **Name (please print):**  Mr Athanasios Saratzis |  |
| **Principal Investigator:** | |
| Signature: | Date: |
| Name: (please print): |  |

**KEY STUDY CONTACTS**

| Chief Investigator | Mr Athanasios Saratzis  Glenfield Hospital, Cardiovascular Research Centre  Groby Road, Leicester, LE39QP  Telephone: 01162523190  Email: [as875@leicester.ac.uk](mailto:as875@leicester.ac.uk) |
| --- | --- |
| Trial Manager | Anish Patel(Trial Manager)  Luke Ingram (Senior Trial Manager)  Leicester Clinical Trials Unit (LCTU)  College of Life Sciences  University of Leicester  Maurice Shock Building  University Road  Leicester  LE1 7RH, UK |
| Sponsor | University of Leicester  Research & Enterprise Division  Research Governance Office  Leicester General Hospital  Gwendolen Road  Leicester  LE5 4PW  [RGOsponsor@le.ac.uk](mailto:RGOsponsor@le.ac.uk) |
| Clinical Trials Unit | Leicester Clinical Trials Unit (LCTU)  College of Life Sciences  University of Leicester  Maurice Shock Building  University Road  Leicester  LE1 7RH, UK  **Email**: [evocc@leicester.ac.uk](mailto:easyas@leicester.ac.uk) |
| Funder | National Institute for Health and Care Research (NIHR) Health Technology Assessment Programme (HTA); Reference: NIHR151230 |
| Funder start and end date(s) | START DATE: 01/01/2023  END DATE: 30/09/2028 |
| Protocol Contributors | **Chief Investigator:**  Athanasios Saratzis - co-author and trial design  **Investigators:**  Alun Davies - co-author, trial design  Athanasios Diamantopoulos - co-author, trial design, lead for interventional radiology involvement  Robert SM Davies - co-author, trial design, lead for vascular surgery involvement  David Mark Epstein - co-author, trial design, lead health economist  Marcus Jepson - co-author, trial design, lead for recruitment support  Daniel Perez - co-author, trial design  Despoina Apergi - lead for patient involvement  Keith Jonothan Harris - co-lead for patient involvement  Hany Zayed - co-author, trial design  **Leicester Clinical Trials Unit:**  Cassey Brookes - co-author, trial design  Shaun Barber – co-author, trial design |
| Lead Statistician | Dr Shaun Barber, Medical Statistician  Leicester Clinical Trials Unit (LCTU)  Maurice Shock Building  University Road  Leicester  LE1 7RH, UK  Telephone: 0116 229 7129  Email: sb766@leicester.ac.uk |
| Trial Statistician | Ana Suazo Di Paola, Medical Statistician  Leicester Clinical Trials Unit (LCTU)  Maurice Shock Building  University Road  Leicester  LE1 7RH, UK  Telephone: 0116 229 7129  Email: [atsdp1@leicester.ac.uk](mailto:atsdp1@leicester.ac.uk) |
| Bristol Quintet Intervention | Dr Marcus Jepson  Qualitative and Implementation Lead  Canynge Hall, 39 Whatley Road, Bristol, BS8 2PS  Telephone: 0117 331 3930  Email: marcus.jepson@bristol.ac.uk |
| NIHR Portfolio adopted | Yes |

**CONTENTS**

[1 BACKGROUND 12](#_Toc133650110)

[2 RATIONALE 13](#_Toc133650111)

[2.1 Healthcare need and clinical effectiveness 13](#_Toc133650112)

[2.2 Economic impact and cost effectiveness 14](#_Toc133650113)

[2.3 Sustained interest and intent 14](#_Toc133650114)

[2.4 Capacity to generate new knowledge 14](#_Toc133650115)

[2.5 Evidence and literature 14](#_Toc133650116)

[2.6 Evidence supporting the need for the trial and study design based on PPI and staff views 15](#_Toc133650117)

[3 RESEARCH QUESTION, OBJECTIVES AND OUTCOME MEASURES 17](#_Toc133650118)

[3.1 Research question 17](#_Toc133650119)

[3.2 Primary objective 17](#_Toc133650120)

[3.3 Secondary objectives 17](#_Toc133650121)

[3.4 Exploratory objective 17](#_Toc133650122)

[4 Outcome measures 18](#_Toc133650123)

[4.1 Primary outcome 18](#_Toc133650124)

[4.2 Secondary outcomes 18](#_Toc133650125)

[4.3 Exploratory outcomes 19](#_Toc133650126)

[5 TRIAL DESIGN and setting 20](#_Toc133650127)

[5.1 Internal pilot and progression criteria 20](#_Toc133650128)

[5.1.1 Qualitative research by QuinteT 21](#_Toc133650129)

[Phase 1: Understanding recruitment 22](#_Toc133650130)

[Phase 2: Development and implementation of recruitment intervention strategies 23](#_Toc133650131)

[QRI consent processes 23](#_Toc133650132)

[QRI data analysis 24](#_Toc133650133)

[6 Eligibility criteria 25](#_Toc133650134)

[6.1 Main trial criteria 26](#_Toc133650135)

[6.1.1 Inclusion criteria 26](#_Toc133650136)

[6.1.2 Exclusion criteria 26](#_Toc133650137)

[6.2 Communication study (patients) criteria 26](#_Toc133650138)

[6.2.1 Inclusion criteria 26](#_Toc133650139)

[6.3 Communication study (healthcare providers) criteria 26](#_Toc133650140)

[6.3.1 Inclusion criteria 26](#_Toc133650141)

[7 Trial Interventions 26](#_Toc133650142)

[7.1 Open surgery 26](#_Toc133650143)

[7.2 ENDOVASCULAR TREATMENT 27](#_Toc133650144)

[8 TRIAL PROCEDURES 27](#_Toc133650145)

[8.1 Participant identification and recruitment for pilot and main RCT 28](#_Toc133650146)

[8.2 Consent 28](#_Toc133650147)

[8.2.1 Optional consent for linkage of NHS routine clinical datasets 29](#_Toc133650148)

[8.3 Randomisation 30](#_Toc133650149)

[8.4 Trial assessments 30](#_Toc133650150)

[8.4.1 Baseline Visit (Day 0) 30](#_Toc133650151)

[8.4.2 Review of Clinical Events – Day of Discharge, Day 30 and Months 6, 12, 24, 36, 48 and 54 30](#_Toc133650152)

[8.4.3 Biochemistry Assessments – Baseline and Month 6 30](#_Toc133650153)

[8.4.4 Ankle Brachial Pressure Index (ABPI) – Baseline and Months 6, 12, 24, 36, 48, 54 31](#_Toc133650154)

[8.4.5 EuroQOL 5D 5L Questionnaire – Baseline and Months 6 and 12 31](#_Toc133650155)

[8.5 Follow-up 33](#_Toc133650156)

[8.6 Qualitative assessments 33](#_Toc133650157)

[8.7 Withdrawal of participation and consent criteria 33](#_Toc133650158)

[8.8 Assessment and management of risk 34](#_Toc133650159)

[8.9 Pandemic adaptations 34](#_Toc133650160)

[8.10 End of trial 34](#_Toc133650161)

[9 SAFETY REPORTING 35](#_Toc133650162)

[9.1 Definitions 35](#_Toc133650163)

[9.2 Reporting procedures for All serious Adverse Events 37](#_Toc133650164)

[9.2.1 Reporting procedures for Adverse Events 37](#_Toc133650165)

[9.2.2 Reporting Procedures for Serious Adverse Events 37](#_Toc133650166)

[9.2.3 Reporting Urgent Safety Measures 38](#_Toc133650167)

[10 ROLES AND RESPONSIBILITIES OF TRIAL MANAGEMENT COMMITEES/GROUPS & INDIVIDUALS 39](#_Toc133650168)

[10.1 Trial Steering Committee (TSC) 39](#_Toc133650169)

[10.2 Data Monitoring Committee (DMC) 39](#_Toc133650170)

[10.3 Trial Management group 40](#_Toc133650171)

[11 STATISTICS AND DATA ANALYSIS 41](#_Toc133650172)

[11.1 Sample size calculation 41](#_Toc133650173)

[11.2 Planned recruitment rate 41](#_Toc133650174)

[11.3 Statistical analysis plan 42](#_Toc133650175)

[11.4 Summary of baseline data 42](#_Toc133650176)

[11.5 Primary outcome analysis 42](#_Toc133650177)

[11.6 Secondary outcome analysis 42](#_Toc133650178)

[11.6.1 Subgroup analyses 43](#_Toc133650179)

[11.6.2 Interim analysis and criteria for the premature termination of the trial 43](#_Toc133650180)

[11.6.3 ADVERSE EVENTS REPORTING 43](#_Toc133650181)

[11.6.4 Procedure(s) to account for missing or spurious data 43](#_Toc133650182)

[11.6.5 Economic evaluation 44](#_Toc133650183)

[12 DATA MANAGEMENT 45](#_Toc133650184)

[12.1 Data flow diagram 45](#_Toc133650185)

[12.2 Data handling and record keeping 45](#_Toc133650186)

[12.2.1 National Data Opt-out 45](#_Toc133650187)

[12.2.2 Data Collection Tools and Source Document Identification 45](#_Toc133650188)

[12.2.3 Data Handling and Record Keeping 47](#_Toc133650189)

[12.3 Access to Data 48](#_Toc133650190)

[12.4 Archiving 48](#_Toc133650191)

[13 MONITORING, AUDIT & INSPECTION 50](#_Toc133650192)

[14 ETHICAL AND REGULATORY CONSIDERATIONS 51](#_Toc133650193)

[14.1 Research Ethics Committee (REC) review AND reports 51](#_Toc133650194)

[14.2 Peer review 51](#_Toc133650195)

[14.3 Public and Patient Involvement (PPI) 52](#_Toc133650196)

[14.4 Regulatory Compliance 54](#_Toc133650197)

[14.5 Protocol compliance 54](#_Toc133650198)

[14.6 Data protection and patient confidentiality 54](#_Toc133650199)

[14.7 Financial 55](#_Toc133650200)

[14.8 Indemnity 55](#_Toc133650201)

[14.9 Post trial care 55](#_Toc133650202)

[14.10 Access to the final trial dataset 55](#_Toc133650203)

[15 DISSEMMINATION POLICY 57](#_Toc133650204)

[16 references 58](#_Toc133650205)

[17 Appendices 59](#_Toc133650206)

[17.1 APPENDIX 1 - Amendment History 59](#_Toc133650207)

[17.2 APPENDIX 2 – data flow diagram 61](#_Toc133650208)

**LIST OF ABBREVIATIONS**

| AE | Adverse Event |
| --- | --- |
| AR | Adverse Reaction |
| BSIR | British Society of Interventional Radiology |
| CDMS | Clinical Data Management System |
| CI | Chief Investigator |
| CLTI | Chronic Limb-Threatening Ischemia |
| CRF | Case Report Form |
| CTU | Clinical Trials Unit |
| GCP | Good Clinical Practice |
| ICF | Informed Consent Form |
| ISF | Investigator Site File |
| JLA | James Lind Alliance |
| LCTU | Leicester Clinical Trials Unit |
| MDT | Multi-Disciplinary Team |
| NHS R&D | National Health Service Research & Development |
| PAD | Peripheral Arterial Disease |
| PI | Principal Investigator |
| PIC | Participant Identification Centre |
| PIS | Participant Information Sheet |
| QC | Quality Control |
| QRI | QuinteT Recruitment Intervention |
| RFS | Research Filestore Service |
| RCT | Randomised Control Trial |
| REC | Research Ethics Committee |
| SAE | Serious Adverse Event |
| SAR | Serious Adverse Reaction |
| SEAR | Screened, Eligible Assess, Randomised |
| SOP | Standard Operating Procedure |
| TASC | Trans-Atlantic Inter-Society Consensus |
| TMF | Trial Master File |
| TMG | Trial Management Group |
| TSC | Trial Steering Committee |
| VSGBI | Vascular Society of Great Britain and Ireland |

**KEY WORDS**

Peripheral arterial disease, endovascular, vascular

**TRIAL SUMMARY**

| **Trial Title** | A randomised controlled trial assessing the clinical and cost-effectiveness of Endovascular vs. Open revascularisation in severe oCClusive aorto-iliac disease. (**EVOCC)** | |
| --- | --- | --- |
| **Trial Design** | A parallel group, multicentre, prospective, open label, randomised controlled trial (with an internal pilot) of endovascular vs. open revascularisation in patients with severe oCClusive aorto-iliac disease. | |
| **Participating Sites:** | Over 30 NHS hospital sites in the UK (England, Scotland and Wales) | |
| **Trial Participants** | Adult patients with symptomatic peripheral arterial disease who have severe aorto-iliac occlusive disease (TASC II class C or D) and are undergoing revascularisation of their aorta/iliac arteries | |
| **Planned Sample Size** | 628 | |
| **Recruitment Period** | 6 month pilot recruitment phase and 24 months for the main recruitment phase. | |
| **Follow Up Duration** | Minimum 2 years (maximum 4.5 years, median 3 years) | |
| **Planned Trial Period** | **Grant start date**: 01/01/2023  Total grant duration 69 months, including 6 month pilot recruitment, 24 months main recruitment and 24 months of patient follow-up. | |
|  | **Objectives** | **Outcome Measures** |
| **Primary** | Establish whether the clinical and cost-effectiveness of open surgery for severe occlusive aorto-iliac disease (TASC II C/D) is superior to endovascular treatment? | Combined measure of death or major lower limb amputation, measured in days from randomisation until first event or until the end of the trial (minimum 2 years) where no event occurs |
| **Secondary** | (i) Identify whether open surgery for severe occlusive aorto-iliac disease is superior to endovascular treatment in terms of amputation-free survival with median 3 year follow-up (minimum 2 years).  (ii) Establish the cost-effectiveness of the interventions including differences in quality-of-life of patients. | (i) Mortality (all cause)  (ii) Cardiovascular events  (iii) Hospital admissions (incl. reason for admission)  (iv) Re-interventions (incl. nature of re-intervention)  (v) All lower limb amputations (minor and major i.e. below and above ankle joint) all measured as time until first event as described for the primary outcome  (vi) Concomitant medications  (vii) Estimated glomerular filtration rate (renal function) based on serum creatinine levels using the Chronic Kidney Disease Epidemiology (CKD-EPI) formula  (viii) Low Density Lipoprotein (LDL)  (ix) HbA1C  (x) Cholesterol  (xi) Ankle Brachial Pressure index (ABPI)  (xii) Details of intervention  (xiii) Quality-of-life using the EQ-5D-5L questionnaire  (xiv) Healthcare resource use and costs across follow-up assessments over a minimum of 24 months |

**ROLE OF TRIAL SPONSOR**

The study has been funded by a grant from the NIHR Health Technology Assessment (HTA) Programme (ref: NIHR151230). Additional support and resources for the study will be provided by the participating Trusts and their corresponding Clinical Research Networks (CRN). The funder will be responsible for funding the study but will not be part of the study conduct, data analysis and interpretation, manuscript writing, and dissemination of results.

The Sponsor, the University of Leicester, will be responsible for all aspects of the study. The Sponsor will delegate duties to other parties, including Leicester Clinical Trials Unit (LCTU), this delegation will be formally documented. However, like the funder, the Sponsor will not be part of the study conduct, data analysis and interpretation, manuscript writing, and dissemination of results.

**TRIAL FLOW CHART**

Setting NHS Hospitals providing specialist care to patients with PAD

Patients with severe occlusive aorto-iliac PAD presenting with symptomatic peripheral arterial disease [rest pain and/or tissue loss (gangrene) and/or life style limiting claudication], already being considered for revascularisation

Screening & enrolment

**Inclusion criteria:** patient deemed eligible for both open & endovascular procedure

**Exclusion criteria:** Age ≤18 years, Asymptomatic PAD

**Randomisation**

**Discussion at vascular meeting**

**Informed consent**

Allocation 1:1

Follow-up

**Open surgery**

314 participants

(With or without inflow/outflow procedures)

**Endovascular treatment**

314 participants

(With or without inflow/outflow procedures)

2-year minimum follow-up including assessments for death, amputation, any subsequent operation, cardiovascular events and quality-of-life (validated questionnaire)

Analysis

Primary outcome of interest: death and/or major lower limb amputation (amputation free survival)

Analysis of clinical & cost-effectiveness

**Internal pilot:**

6 months

10 sites (nationally)

**Main recruitment period:**

24 months

Over 30 sites (nationally)

628 participants

**Figure 1. EVOCC CONSORT diagram**

Figure 1

# BACKGROUND

The clinical and cost-effectiveness of open surgery vs. minimally invasive endovascular treatment for severe occlusive aortic and iliac artery disease is unknown. This area has been identified as a research priority in a recent James Lind Alliance (JLA) process across several vascular disease areas, such as peripheral arterial disease (PAD), diabetic foot care, and amputations (1).

Lower limb Peripheral Arterial Disease (PAD) affects 20% of people over 55 years of age (2) and is the main cause of amputations in the NHS (2). Often, patients with PAD develop leg pain when walking (called claudication). Some patients might also develop leg pain at rest or lower limb necrosis, a condition called Chronic Limb Threatening Ischaemia (CLTI) (3). This is limb and life threatening and requires urgent revascularisation to save the patient’s limb/life (3). The aorta and iliac arteries are common sites affected in people with lifestyle limiting claudication or CLTI (4). These patients can have revascularisation either with open surgery or more recently developed minimally invasive techniques in the form of endovascular treatment (3). Both open surgery and endovascular treatment, however, are associated with significant complications. Open surgery is associated with a 3% early mortality and many perioperative complications (5). Endovascular treatment is less invasive, with a 1% early mortality rate (5, 6). Endovascular devices, however, are costly and 43% of patients need re-intervention within 3 years (5).

Patients in our study development focus groups expressed a preference for endovascular treatment. They were, however, concerned about the many re-interventions and high number of late deaths or amputations potentially associated with endovascular treatment. Some patients even voiced concerns about the high cost of the endovascular devices and many re-interventions. There is no randomised evidence comparing the clinical and cost-effectiveness of these techniques in this context, potentially leading to more deaths, amputations, and increased NHS costs. Clinicians therefore do not know which treatment to offer this cohort. The need to improve outcomes and reduce amputations in CLTI/claudication has been highlighted by the JLA and Vascular Society of Great Britain and Ireland (VSGBI) in 2021 as a key research priority (1).

# RATIONALE

There is no randomised evidence comparing the clinical and cost-effectiveness of open or endovascular interventions to address severe occlusive aorto-iliac disease, which creates great uncertainty for patients with PAD and clinicians. It also leads to an estimated 7,000 excess deaths and amputations, and £180 million in excess NHS costs per year, based on our published evidence and HES data (6, 7). The need to improve outcomes and reduce amputations in this group of patients has been highlighted by the JLA in 2021 as an urgent research priority (1).

## Healthcare need and clinical effectiveness

An international multidisciplinary expert group in PAD, the Trans-Atlantic Inter-Society Consensus (TASC) force, classified aorto-iliac PAD based on severity of arterial stenoses or occlusions from grade A (less severe) to grade D (most severe) (1). For TASC A/B disease (1), endovascular treatment is the universally established and accepted strategy both in the NHS and globally (1). For TASC C/D disease, there is great uncertainty regarding the effectiveness of open surgery vs. endovascular treatment, reflected in all guidelines (1). The fact that endovascular therapies (stents, angioplasties, other endovascular procedures) have seen great advances in the last 20 years has added even more uncertainty regarding the management of patients with TASC C/D aorto-iliac PAD. Clinicians and patients do not know whether surgery or endovascular treatment, especially with the newer devices now available across the NHS as routine care, are better in terms of saving lives, legs, and improving quality of life.


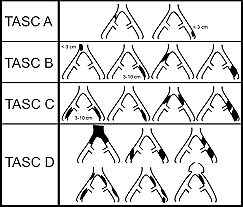


**Figure 2.** TASC classification for patients with aorto-iliac arterial disease. TASC C and D patients have severe occlusive disease and are more likely to present with severe symptoms; these patients will be eligible to take part in EVOCC as there is treatment uncertainty in this group regarding superiority of surgery over newer endovascular (minimally invasive) treatment.

## Economic impact and cost effectiveness

We obtained information from our published meta-analysis (1) and HES to identify patients with CLTI/claudication and TASC C/D aorto-iliac disease who had revascularisation between 2014-2019. We then constructed an early decision model to calculate 3 year death, amputation, and re-intervention rates, NHS resource use, and quality-adjusted life years (QALYs). Open surgery is associated with a peri-operative mortality of 3% vs. 1% for endovascular treatment. The latter is associated with more re-interventions and late amputations/deaths. Three year survival is 70% for open surgery vs. 65% for endovascular treatment (HES data). Open surgery costs arise from intensive care stay and early complications. Endovascular treatment costs arise from devices, re-interventions, and late amputations. Average peri-operative and device costs are £13,765 for endovascular treatment. Late re-interventions and amputations cost £6,811, totalling £20,576 for endovascular treatment over 3 years. Open surgery costs £10,675 over 3 years (£9,901 less per patient). The 3 year QALY difference is 0.153, favouring open surgery, despite the higher immediate mortality/morbidity. All of these estimates, however, are based on unadjusted observational data. Overall, it is very uncertain whether endovascular treatment, despite being minimally invasive, is cost-effective in the NHS.

## Sustained interest and intent

PAD is very common, especially in those with diabetes, socioeconomically deprived populations, South Asian, Afro Caribbean, and Eastern European minorities (1), with increasing prevalence nationally (2,3). Symptomatic PAD, mostly in the form of CLTI or lifestyle limiting claudication, accounts for 75% of the current NHS vascular workload based on HES, the National Vascular Registry, and cohort studies (1). The number of amputations due to PAD is rising, especially in those with diabetes (1).

## Capacity to generate new knowledge

This will be the first randomised trial assessing clinical and cost-effectiveness in this clinical context worldwide.

## Evidence and literature

We published a meta-analysis of 66 observational studies (9,319 patients) (1) and a cohort study of patients having aorto-iliac endovascular treatment across multiple NHS hospitals (1). We also identified all patients who had surgery or endovascular treatment (2014 to 2019) for TASC C/D aorto-iliac disease using HES and recorded 3 year outcomes. We performed an audit in 7 NHS hospitals to identify how many patients treated for TASC C/D aorto-iliac disease are eligible for randomisation. We have searched all trial repositories including the WHO trials registry in December 2021 (updated April 2022). Main findings:

- There are no relevant completed or ongoing trials; a recently completed trial in a similar context did not include all-comers and aorto-iliac disease, but only selected patients with iliac and common femoral artery disease, treated with a combination of open surgery and endovascular devices (1)
- Between 2014-2019, an average 2,200 patients per year received open surgery for these indications and 6,800 received endovascular treatment in the NHS (HES)
- Peri-operative mortality in observational series and HES, not adjusted for co-morbidities or anatomical complexity, was 3% for open surgery vs. 1% for endovascular treatment; 43% of those having endovascular treatment required re-intervention within 3 years
- In the meta-analysis, we observed significant bias favouring publication of studies with positive outcomes
- In the audit of 7 NHS centres, 82% of patients with TASC C/D disease would be eligible for randomisation in EVOCC
- No relevant PPI initiatives were identified in the literature
- Healthcare professionals’ views on equipoise had not been explored.

## Evidence supporting the need for the trial and study design based on PPI and staff views

Three patient focus groups were convened to develop EVOCC. Eleven patients who had endovascular treatment, 7 who had open surgery, 3 carers, and 4 partners took part, from multiple ethnic and socioeconomic backgrounds. Most patients had diabetes and 5 previously had a leg amputation. We explored outcomes of interest, follow-up procedures, study acceptability, and clinically important differences. All agreed that the main outcome measure should include mortality and amputations (equally weighted), which are the two things that matter most to patients. They specifically supported that follow-up should continue for a minimum of 2 years. Patients reported that they keep needing re-interventions or amputations several months after surgery and/or endovascular treatment. All would take part in the study and found randomisation acceptable. When asked, they supported a superiority design of surgery vs. endovascular treatment. Patients would like to know whether the more invasive option (surgery) is better at saving legs and lives vs. the least invasive treatment. These patients have reviewed and approved the study design.

To explore equipoise amongst healthcare staff nationally, we performed a survey of 60 surgeons, 23 radiologists, and 8 nurses. This covered 38 NHS sites (64% of all UK vascular centres). Overall, 97% agreed this trial is necessary; 88% felt it would be possible to deliver within a 2 year main recruitment period. Finally, 91% of surgeons and radiologists felt they were in equipoise and would recruit/randomise patients.

# RESEARCH QUESTION, OBJECTIVES AND OUTCOME MEASURES

## Research question

Is the clinical and cost-effectiveness of open surgery for severe occlusive disease of the aorta and iliac arteries superior to newer endovascular (keyhole) treatment?

## Primary objective

To identify whether the clinical and cost-effectiveness of open surgery for severe occlusive disease (TASC II C/D) of the aorta and iliac arteries is superior to endovascular treatment.

## Secondary objectives

- Identify whether open surgery for severe occlusive aorto-iliac disease is superior to endovascular treatment in terms of amputation-free survival (death and/or major lower limb amputation) over a minimum 2 year follow-up period (median 3 years).
- Establish the cost-effectiveness of the two interventions, including quality-of-life differences.

## Exploratory objective

To identify any association between the use of different endovascular techniques or open surgical configuration and subsequent primary outcome (amputation free survival).

# Outcome measures

## Primary outcome

Combined measure of death or major lower limb amputation, measured in days from randomisation until first event or until the end of the trial (minimum 2 years) where no event occurs.

## Secondary outcomes

Secondary outcome measures are:

1. Mortality (all cause), measured in days from randomisation until first event or until the end of the trial (minimum 2 years) where no event occurs
2. Cardiovascular events (myocardial infarction, stroke, admission for heart failure), measured in days from randomisation until first event or until the end of the trial (minimum 2 years) where no event occurs
3. Number of hospital admissions (including reasons for readmissions), following discharge from index surgical admission for up to 4.5 years
4. Number of re-interventions (including nature of re-intervention), following index surgical procedure for up to 4.5 years
5. All lower limb amputations (minor and major i.e. below and above ankle joint) measured in days from randomisation until first event or until the end of the trial (minimum 2 years) where no event occurs
6. Concomitant medications (specifically: use of statin, use of antithrombotic medication)
7. Estimated glomerular filtration rate (renal function) based on serum creatinine levels using the Chronic Kidney Disease Epidemiology (CKD-EPI) formula measured at 6 and 12 months and annually thereafter
8. Low Density Lipoprotein measured at 6 months
9. HbA1C measured at 6 months
10. Cholesterol measured at 6 months
11. Ankle Brachial Pressure Index (ABPI) measured at 6 and 12 months and annually thereafter
12. Types of endovascular devices used and/or name of surgery performed (open)
13. Quality-of-life using the EQ-5D-5L measured at 6 and 12 months and annually thereafter
14. Healthcare resource use and costs measured across follow-up assessments over a minimum of 24 months and until the end of the trial

A prospectively planned economic evaluation will be conducted from an NHS and personal social services perspective as per NICE recommendations.

## Exploratory outcomes

To identify any association between the use of different endovascular techniques or open surgical configuration and subsequent outcomes (amputation free survival).

# TRIAL DESIGN and setting

The EVOCC trial is a two-arm prospective, superiority, multicentre (national), randomised controlled trial (RCT) comparing two modes of treatment which are already routine NHS care, in over30 UK sites (NHS hospitals which already provide care for PAD to patients). Open surgery will be compared to the newer treatment (intervention) using established methodology and pre-specified plans by the CTU team and statisticians. This will address the existing uncertainty whether surgery is superior (clinical and cost-effectiveness) to endovascular treatment. Blinding is impossible in this setting given the completely different nature of the two trial treatments.

Thirty NHS hospital vascular networks which routinely provide care to patients with PAD have confirmed they will take part, across a wide geographical spread (urban and rural); the sites treat a diverse (ethnically and socioeconomically) PAD population, with high prevalence of amputations and diabetes.

The target population is individuals ≥18 years with severe occlusive aorto-iliac disease (TASC II C or D class) presenting with ischaemic lower limb rest pain and/or tissue loss and/or lifestyle limiting claudication to NHS vascular hospital networks. Patients will be screened and recruited across all secondary care settings in those hospitals (clinic, ward, on-call, remote referrals). Randomisation will take place as soon as the patient provides written informed consent. Patients undergoing these treatments in the NHS are usually discussed at multidisciplinary (MDT) team meetings. The patients will be approached for consent once the MDT feel are in equipoise regarding the treatments.

## Internal pilot and progression criteria

Given the complexities relating to the recruitment of individuals with symptomatic PAD, an internal pilot phase has been planned based on NIHR guidance, national surveys, audits and HES data. The progression criteria have been calculated based on the requirement to recruit 628 eligible patients from 30 sites during a 30 month recruitment period (including the internal pilot involving 10 sites). Recruitment rates and progression criteria have been based on completed NIHR HTA trials recruiting patients with symptomatic PAD. Assuming a very conservative ~6% consent rate, the aim is to recruit at least 1 participant per site per month during the pilot phase and subsequently in the main trial phase.

**Proposed pilot length**: The 6 month internal pilot will commence following a 6 month trial set up and regulatory approval period and 3 month period for pilot site set up (overall 9 months for set up). At least 10 sites will open to recruitment for the 6 month duration of the internal pilot. This will also allow the qualitative Bristol QuinteT team to conduct interviews and qualitative assessments at each of these sites. Upon successful recruitment and site set ups, the trial will progress to recruit from over 30 sites for an additional 24 months. The Trial Steering Committee (including PPI partners and CTU representation) will meet at the end of the pilot recruitment period (project month 16) to assess progression criteria, summarised in Table 1.

**Progression criteria:** Green, proceed to main phase (100% of all targets achieved); Amber, reflect, remodel, and adjust project plan in consultation with funder [75%-99% (minimum) of all targets achieved]; Red, trial stopped (<75% of any target achieved).

**Table 1. Internal Pilot progression criteria**

| **Progression criteria** | **Red** | **Amber** | **Green** |
| --- | --- | --- | --- |
| **% Threshold** | <74% | 75-99% | **100%** - all below criteria must be met to proceed directly to main recruitment phase. |
| **Recruitment rate/site/month** | ≤ 0.74 participants per month per site | 0.75 to 0.99 participant per month per site | ≥ 1.0 participant per month per site |
| **Number of sites opened by the end of the pilot phase** | ≤ 6 | 7 to 9 | ≥10 |
| **Total number of participants recruited** | ≤ 44 across all pilot sites over 6 months | 45 to 59 across all pilot sites over 6 months | ≥ 60 across all pilot sites over 6 months |

### Qualitative research by QuinteT

We will optimise recruitment with the inclusion of a QuinteT Recruitment Intervention (QRI) led by qualitative experts (based at the University of Bristol). The overarching aim of the QRI is to support recruitment to EVOCC. Initially, the QRI team will share existing knowledge of approaches that help with information delivery and trial conduct. Thereafter, it will employ the QRI methodology: a mixed-methods approach that has been implemented in over 30 RCTs to optimise recruitment and informed consent (52). The QRI employs qualitative and mixed-method approaches to understand recruitment issues rapidly (Phase 1), and then uses this evidence to design and implement tailored strategies to optimise recruitment processes (Phase 2). The QRI will be integrated in the pilot phase and learning from this will be transferred to the main RCT. Further information on Phase 1 and Phase 2 can be found below.

**Phase 1: Understanding recruitment**

Phase I aims to understand trial recruitment processes and how these may differ across participating sites. It will specifically aim to support staff in overcoming challenges in recruiting participants from certain minorities, deprived backgrounds, and those who are frail. A multi-faceted, flexible approach will be employed, including one or more of the following:

1. Semi-structured interviews with: (i) members of the TMG (ii) clinicians or researchers involved in trial recruitment (‘recruiters’) (n=10-25), and (iii) eligible patients who have been approached to take part in the trial (n=5-15). Interviews with members of the TMG and recruiters will focus on: their perspectives on the evidence upon which the trial is based; perceptions of uncertainty/equipoise; views on the appropriateness of eligibility criteria, and (where relevant) experiences of recruitment difficulties or successes. Interviews with patients will explore views on the presentation of trial information, understandings of trial processes (e.g., randomisation), and reasons underlying decisions to accept or decline the trial. Patients will be purposefully sampled, to build a sample of maximum variation based on age, gender, trial centre, and their decision about trial participation (i.e., accept or decline). Interviews will be conducted via telephone or a secure web-conference facility (e.g., Skype, MS Teams, Zoom or Google Hangouts), where conducting these in person is not feasible.
2. Analysis of audio-recorded recruitment discussions: ‘Recruitment consultations’ between recruiters and potential participants will be audio-recorded with permission. This will be a core aspect of data collection, as it allows an opportunity to investigate actual (rather than perceived) recruitment behaviours. The audio recordings will be used to examine how the trial treatments and trial processes (e.g., randomisation) are explained to patients, how equipoise is conveyed, and how patients react to the information provided. Audio-recordings of recruiter-patient consultations do not require the presence of a researcher. If recruitment consultations need to be conducted remotely via telephone due to COVID-19 or other restrictions, equipment will be provided to allow the recruiters to audio-record their telephone calls with patients (upon receipt of appropriate consent).
3. Scrutiny of trial screening logs and mapping of recruitment pathways: working in close collaboration with LCTU, a comprehensive screening log will be designed to capture numbers of patients screened, eligible, approached, randomised, and numbers accepting their randomised allocation. The logs and interviews (described above) will be used to construct flow charts depicting the ‘recruitment pathway’ for each centre, and the points at which patients enter/exit the pathway. Numbers captured in the screening logs will be compared across centres and considered in relation to estimates specified in the grant application/trial protocol. This process will be regularly repeated to identify bottlenecks in the recruitment pathway and inform further data collection through interviews and/or analysis of consultations.

**Phase 2: Development and implementation of recruitment intervention strategies**

Working closely with the CI and TMG, a ‘plan of action’ will be formulated, consisting of specific strategies designed to improve recruitment and informed consent processes. The plan will be informed by Phase 1 evidence and is likely to include generic and site-specific interventions. Generic interventions may include written guidance documents that provide suggestions on how to explain the trial to patients in a balanced way (i.e. conveying equipoise). Supportive feedback will feature heavily in the plan of action, with the precise nature and timing dependent on the issues that arise. For example, feedback may be offered to individual centres, through multi-centre feedback or confidentially to individual recruiters. All feedback will be supported with anonymised data extracts from interviews and recruitment consultations.

Although the QRI has been presented as two distinct phases for clarity, the phases will overlap. New avenues of enquiry will arise as data collection proceeds and new centres open, and the feedback sessions themselves may highlight new issues that warrant further investigation. Screening logs will be assessed throughout the recruitment period, with particular scrutiny of figures before/after Phase 2 interventions are implemented. This will help to inform decisions about further investigation or intervention throughout the trial’s recruitment period.

**QRI consent processes**

Healthcare professionals (HCPs) involved in the EVOCC trial recruitment will receive a copy of the ‘QRI healthcare professional information sheet’ at Site Initiation Visits (SIVs) or via email from the QRI researcher. This will explain the QRI processes described above (specifically, audio-recording of recruitment discussions and interviews). Research nurses or the QRI researcher will obtain written consent from HCPs using a consent form. HCPs may opt to participate in just one, both, or neither of the QRI elements.

Patients will also receive a ‘QRI Participant information sheet’ (PIS) explaining the audio-recording of recruitment discussions and the possibility of being approached for a future interview. The PIS will be handed to patients during the first discussion about the RCT. As we wish to capture how the RCT is introduced to patients, we will employ a two-step consent process for audio-recording recruitment discussions. In brief:

- A member of the clinical team (who by default, may also be a member of the research team) will obtain verbal consent to record the initial discussion about EVOCC. If patients agree, the ‘verbal consent form’ will be signed by the HCP to document that verbal consent has been obtained, and the discussion will be recorded.
- Patients will receive the QRI PIS in the first consultation, and will be provided sufficient time to ask any questions and consider their participation in the QRI.
- Patients will be asked to provide written informed consent for the audio-recordings and/or interviews in their subsequent baseline visit. Further RCT recruitment discussions will be audio-recorded subject to receiving written consent; if written consent is not obtained, the recording captured from the first discussion will be deleted (if collected) and no further recordings will be made.

Patients can accept or decline participation in the audio-recordings, interviews, or both. Their decision(s) about QRI participation will be independent to their decision about RCT participation.

A verbal consent process will also be employed to reduce burden on HCP participants, where obtaining consent in person is problematic (e.g., due to lack of research nurse capacity). Potential participants will be sent a copy of the study information sheet and consent form via email. The QRI researcher will call the HCP and read each statement on the consent form, initial these as is appropriate, and sign to confirm they have obtained consent. The consent discussion will be audio-recorded and a copy of the completed form will be sent to the participant for their records. All QRI consent forms will be retained at sites.

In light of the COVID-19 pandemic, all of the consent processes for the QRI can be easily adapted to ‘verbal consent’ processes, whereby the individual taking consent reads the consent form statements and signs on the potential participant’s behalf. These discussions can be audio-recorded to provide a record of the potential participant confirming their consent. These verbal consent processes will likely take place irrespective of whether the consultation is taking place in person or remotely, in an effort to minimise risk of transmission (e.g., through use of pens, contact with paper, etc).

**QRI data analysis**

All qualitative interviews will be audio-recorded using digital encrypted recorders, transcribed verbatim, and edited to ensure anonymity. Audio-recordings will be transcribed by internal University of Bristol staff or an external transcription company which has signed the necessary University of Bristol confidentiality agreements. Transcripts will be pseudo-anonymised. Interview data will be managed using NVivo software (QRS International) and analysed thematically using constant comparative approaches adopted from Grounded Theory. Audio-recorded recruitment consultations and follow up discussions will be subjected to content, thematic, and novel analytical approaches, such as targeted conversation analysis. There will be a focus on aspects of information provision that are unclear, disrupted, or potentially detrimental to recruitment and/or adherence. Standard approaches to enhancing rigour, such as double-coding, triangulating, and seeking out ‘negative cases’, will be employed throughout.

# Eligibility criteria

## Main trial criteria

### Inclusion criteria

- Age ≥18 years (no upper age limit)
- Presence of symptomatic PAD (tissue loss and/or rest pain and/or lifestyle limiting claudication)
- Severe aorto-iliac occlusive PAD defined as “Inter-Society Consensus for the Management of Peripheral Arterial Disease” (TASC II) class C or class D diagnosed on prior cross-sectional imaging
- Patient discussed in any local vascular Multi-Disciplinary Team (MDT) meeting/discussion and deemed suitable for open surgery or endovascular treatment.
- Patient willing and able to give consent and commit to study follow-up.
- An ability to understand written English or availability of a translator to explain the trial documentation

### Exclusion criteria

Asymptomatic PAD

## Communication study (patients) criteria

### Inclusion criteria

- Patient willing and able to give consent
- An ability to understand written English or availability of a translator to explain the study documentation
- Involved with research visits with nurses and/or doctors that have provided consent for the HCP Communication Study (where related to audio-recording of research visits)

## Communication study (healthcare providers) criteria

### Inclusion criteria

- Willing and able to give consent
- Involved with research visits with patients that have provided consent for the Communication Study (where related to audio-recording of research visits)

**PLEASE NOTE: Participants must meet all eligibility criteria to be considered for the EVOCC trial. There are NO eligibility waivers.**

# Trial Interventions

## Open surgery

Open surgical revascularisation is an established treatment strategy for patients with symptomatic PAD and aortoiliac occlusive disease in the NHS. The exact configuration of the open surgical revascularisation will be left to the clinicians who offer the treatment at each site. Such procedures include aorto-(bi/uni)-femoral/iliac bypass, femoro-femoral crossover, axillo-femoral bypass (unilateral or bilateral), aortic endarterectomy, iliac endarterectomy (unilateral or bilateral) or a combination of these procedures. Inflow and outflow procedures, both open and endovascular, will be allowed and left at the discrepancy of the treating clinicians/centres (e.g., concomitant endarterectomy or any form of adjunct endovascular procedure deemed necessary locally).

## ENDOVASCULAR TREATMENT

Endovascular treatment is an established treatment strategy for patients with symptomatic PAD and aortoiliac occlusive disease in the NHS. The exact type of procedure offered to each patient will be decided locally, based on clinicians’ preferences and patient characteristics. Any type of angioplasty device, stent, vessel preparation strategy, or other adjunct endovascular procedure can be used, at the discretion of the clinicians at each trial site. Inflow and outflow procedures, including open surgical access to arteries or open surgical reconstruction to ensure adequate inflow/outflow will be allowed.

For both open surgery and endovascular treatment, inflow and outflow procedures (e.g. adjunct open surgery when undergoing endovascular treatment or vice versa) will be left to the treating clinicians to decide/offer.

# TRIAL PROCEDURES

## Participant identification and recruitment for pilot and main RCT

The Trial Schema () demonstrates the recruitment process in addition to the treatments and follow-up schedule. The recruitment phase will commence as soon as there is ethical, research governance and regulatory approval, local site capacity and capability and only after the Sponsor green light has been issued.

Potentially eligible participants are identified by the clinical care team through surgical waiting lists, clinics (elective or emergency), on calls, or by the patient’s direct clinical care team; they will be discussed at vascular team meetings to confirm eligibility and suitability to take part in the trial. This allows staff to identify patients with consent capacity issues early.

Following identification by the clinical team, the patients will be approached by a delegated member of the clinical team. The potential participant will be provided with the Participant Information Sheet (PIS) either in person, by post, or via e-mail (as per their preference).

## Consent

The Principal Investigator (PI) retains overall responsibility for the conduct of research at their site, this includes the taking of informed consent of participants at their site. They must ensure that any person delegated responsibility to participate in the informed consent process is duly authorised, trained and competent to participate according to the ethically approved protocol, principles of Good Clinical Practice (GCP) and Declaration of Helsinki. If delegation of consent is acceptable then details should be recorded in the Delegation of Authority Log.

Telephone, electronic or written informed consent must be obtained prior to the participant undergoing procedures that are specifically for the purposes of the trial and are out-with standard routine care at the participating site (including the collection of identifiable participant data).

The right of a participant to refuse participation without giving reasons must be respected.

The option of electronic consent and telephone consent has been developed as an alternative to face-to-face methods. This model has been developed where it is not in the best interests of the patient to bring them back to hospital for research purposes, the patient does not want to or are unable to come into the hospital. It is anticipated that the majority of potential participants will wish to have a face-to-face visit. The electronic consent process will mirror the content of the paper documents, including an electronic PIS, and will be supported by the REDCap platform provided by Biomedical Research Centre Health Informatics. This will be supported by a Video/Telephone Consent Form to be completed by the researcher or delegated site person performing consent, confirming that the participant has understood the information and consent process. The electronic consent system will only be accessible by appropriate personnel active on the study who will only be able to view consents for their site. The system will notify of any errors in a submitted consent form which can be rectified by the participant, as well as produce a PDF for the participant, research and medical records. Where appropriate, electronic consent will be supported either over the telephone or by video conference between the participant and the clinician/researcher. The remote consent process will also mirror the content of the paper documents. The Video/Telephone Consent Form will be completed by the researcher or delegated site person receiving informed consent, confirming that the participant has understood the information and has capacity to consent. The remote consent process will only be performed by delegated personnel at each site.

The participant must remain free to withdraw at any time from the trial without giving reasons and without prejudicing his/her further treatment and must be provided with a contact point where he/she may obtain further information about the trial. Data collected up to the point of withdrawal will be retained and used. Where a participant is required to re-consent or new information is required to be provided to a participant it is the responsibility of the PI to ensure this is done in a timely manner.

A Participant Information Sheet (PIS) will be provided to facilitate this process. The Principal Investigator or their delegate will ensure that they adequately explain to the participant the aim, trial intervention, anticipated benefits and potential risks of taking part in the trial.

The participant will be given time to consider the information, and the opportunity to question the Investigator, their GP or other independent parties to decide whether they will participate in the trial. They will be given sufficient time, usually 24 hours, to make their decision. Written Informed Consent will then be obtained by means of participant dated signature and dated signature of the person who presented and obtained the informed consent. The person who obtained the consent must be suitably qualified and experienced, and have been authorised to do so by the Principal Investigator as detailed on the Delegation of Authority and Signature log for the trial. The original signed form will be retained at the trial site within the Investigator Site File (ISF). A copy of the signed Informed Consent will be given to participants and a copy retained in the participant medical notes. The outcome of the conversation, i.e., provided informed consent and have been randomised to the trial, will be recorded in the trial screening log. This will be necessary for assessing recruitment feasibility.

The ICF contains optional items relating to consent for the use of data held by central NHS bodies (see below) and the retention of contact information to enable future contact for consideration of participation in follow-up studies. Participants may provide or decline consent for any of these items independently. Should new and important information, particularly relating to safety, become available during the trial, participants may be required to re-consent to the trial via an updated PIS and ICF.

Once the participant is randomised onto the trial, their Participant ID Number will be populated and recorded on the ICF and all Case Report Forms (CRFs) going forward.

Details of the informed consent discussions should be recorded in the participant’s medical notes in accordance with Good Clinical Practice (GCP). This should include date of discussion, the name of the trial, outcome of the discussion, version number of the PIS given, version number of ICF signed and date consent received.

### Optional consent for linkage of NHS routine clinical datasets

EVOCC includes an optional consent to allow linkage to patient data available in NHS routine clinical datasets, such as the Hospital Episode Statistics (HES) and mortality data from the Office of National Statistics (ONS) through The Health and Social Care Information Centre and other central UK NHS bodies. This consent will also allow access to other new central UK NHS databases that may appear in the future so that relevant long-term outcomes and health resource usage data can be recorded to extend the follow-up of participants without needing to contact them further. This is important as it will link treatments that may become a clinical standard of care to long-term outcomes that are routinely collected in clinical data, but which will not be collected during the follow-up period of the trial.

## Randomisation

After the consent form has been signed, the participant will be randomised to receive one of the two arms; open surgery or endovascular treatment. Participants will be randomised via the 24 hour, web-based service, Sealed Envelope.

Random allocations will have a 1:1 ratio, will be minimised by the following variables: age above 65 years, sex, and presence of tissue loss (yes/no) and stratified by site. Randomisation forms should be completed alongside online randomisation. After a participant is randomised, the user is presented with the allocated treatment group and the unique Participant ID Number. A notification email will be sent to the person conducting the randomisation, copying in Leicester Clinical Trials Unit via the EVOCC trial mailbox. The Participant ID number must be entered onto the participant signed ICF and all CRFs from this point forward and will link the participant details to the trial data. Any errors in the randomisation process should be reported to Leicester Clinical Trials Unit as soon as reasonably possible after being identified.

## Trial assessments

### Baseline Visit (Day 0)

Once a Participant ID Number is allocated, the Baseline visit can take place. During this visit, participants will complete participant questionnaires, undergo a review of their medical history (including previous surgical interventions), cardiovascular risk factors, PAD anatomy, eGFR, HbA1C, LDL and cholesterol levels. If a female is thought to be pregnant, a urine pregnancy test will be performed as part of usual care ahead of surgery. Where a female participant is found to be pregnant, progression to treatment will be assessed using a risk-based approach as managed routinely by the patient’s treating clinician. Baseline visit biochemistry assessments/bloods to be taken in line with clinical pathway and matching the research schedule as closely as possible. It is understood that because these bloods are clinical, that there may be occasions where these bloods are taken ahead of the research baseline timepoint. Please refer to **Table 2** for further details about the Baseline visit, follow up visits and assessments.

### Review of Clinical Events – Day of Discharge, Day 30 and Months 6, 12, 24, 36, 48 and 54

A review of clinical events, particularly those related to the cardiovascular system, will be undertaken at the above trial visits including re-intervention, admission to hospital, death, myocardial infarction, stroke and new heart failure leading to admission. For visits at day 30, months 12, 36, 48 and 54, data collection will be performed either by telephoning the participant or retrieving the information from their medical notes. For visit month 6, the participant needs to attend clinic in person. However, if the participant cannot attend in person, as this is a pragmatic study this information can instead be collected remotely via telephone or through acquiring information through participant medical records.

### Biochemistry Assessments – Baseline and Month 6

Standard blood sample assessments to be undertaken in clinic at Baseline and month 6 and should include eGFR, total cholesterol, LDL cholesterol and HbA1C - these are NHS standard of care and constitute part of these patients regular care as per NHS and National Institute for Health and Care Excellence guidance. The blood samples (5ml collected at each visit) will be collected at room temperature before being processed and analysed at the local pathology laboratories as per each site’s standard procedures for these patients.

### Ankle Brachial Pressure Index (ABPI) – Baseline and Months 6, 12, 24, 36, 48, 54

ABPI is a reliable way to detect arterial insufficiency and is the ratio of the ankle systolic blood pressure to the brachial systolic blood pressure. It will be measured using a sphygmomanometer and a hand-held Doppler device by appropriately trained personnel e.g., a research nurse or a clinician. This constitutes part of these patients regular care as per NHS and National Institute for Health and Care Excellence guidance.

An ABPI ratio of:

- < 0.5 suggests severe arterial disease
- > 0.5 to < 0.8 suggests the presence of arterial disease or mixed arterial/venous disease
- Between 08 – 1.3 suggests no evidence of significant arterial disease
- > 1.3 may suggest the presence of arterial calcification

Measurements at 30 days and months 6, 12, 36, 48 and 54 can be taken from the participant’s medical records.

### EuroQOL 5D 5L Questionnaire – Baseline and Months 6 and 12

EuroQOL- 5D (EQ-5D-5L) is a standardised assessment of health status to provide a simple, generic

measure of health for clinical evaluation (10, 11). It consists of 2 pages covering 5 dimensions (mobility, self-care, usual activities, pain/discomfort and anxiety/depression) and the participant is

asked to choose an accompanying statement which best describes how much ease or difficulty they

have in each of these dimensions (1 is scored as no problems/pain/anxiety/depression, 5 is scored as

unable to/extreme anxiety/depression). The questionnaire also asks the participant to indicate on a scale of 1-100 1 = the worst health you can imagine, 100 = the best health you can imagine how their health is on that day.

**Table 2. Schedule of Assessments**

| **VISIT** | **Time points** | | | | | | | | | |
| --- | --- | --- | --- | --- | --- | --- | --- | --- | --- | --- |
|  | **Baseline** | **Trial intervention** | **Day of Discharge** | **30 Days** | **6 months** | **12 months** | **24 months** | **36 months^1^** | **48 months^1^** | **54 months^1^** |
| **Visit window (days)**  (± number of days this visit can take place ahead or after its due date) | - | **-** | **-** | ± 7 days | ± 30days | ± 30 days | ± 30 days | ± 30 days | ± 30 days | ± 30 days |
| **Day** | 0 |  |  | 30 | 180 | 365 | 730 | 1095 | 1460 | 1643 |
| Eligibility check | x |  |  |  |  |  |  |  |  |  |
| Obtain informed consent | x |  |  |  |  |  |  |  |  |  |
| Randomisation (allocation of treatment) | x |  |  |  |  |  |  |  |  |  |
| Demographics | x |  |  |  |  |  |  |  |  |  |
| Medical history including cardiovascular risk factors | x |  |  |  | x |  |  |  |  |  |
| Details of anatomy (arteries) | x |  |  |  |  |  |  |  |  |  |
| Concomitant medications (statins and antithrombotics) | x |  | x | x | x | x | x | x | x | x |
| Review of clinical events |  |  | x | x | x | x | x | x | x | x |
| Days lost from regular activities |  |  |  |  | x | x | x | x | x | x |
| Estimated Glomerular Filtration Rate (eGFR) | x |  |  |  | x |  |  |  |  |  |
| Low Density Lipoprotein (LDL) value | x |  |  |  | x |  |  |  |  |  |
| Total Cholesterol value | x |  |  |  | x |  |  |  |  |  |
| HbA1C value | x |  |  |  | x |  |  |  |  |  |
| Ankle Brachial Pressure Index (ABPI) | x |  |  |  | x | x | x | x | x | x |
| EQ-5D-5L questionnaire | x |  |  |  | x | x | x | x | x | x |
| Details of intervention (type of surgery or endovascular treatment configuration and devices) |  | x |  |  |  |  |  |  |  |  |
| Admission to hospital or visit to primary care |  |  |  |  | x | x | x | x | x | x |
| Assessment of Adverse Events | x | x | x | x | x | x | x | x | x | x |

^1^ The follow up trial visitsmay be undertaken remotely either via telephone or through acquiring information through participant medical records. For 6 months, in the first instance please try to bring the patient in for a face-to-face appointment. If this is not possible then this follow-up visit may also be undertaken remotely via telephone or through acquiring information through participant medical records.

## Follow-up

The minimum follow up period for participants is 24 months after the time of randomisation. All follow up data will be collected from the date of randomisation. For month 6 follow-up, please make all efforts to ensure this takes place in person at the vascular clinic (as part of standard of NHS care appointments post-operatively). From month 12 onwards follow-up can be conducted via telephone or through collecting data from participant medical records. Please refer to **Table 2** for further details about follow up visits.

## Qualitative assessments

The Bristol QuinteT team will conduct interviews and qualitative assessments at each of the sites involved in the internal pilot. The QuinteT team will contact participants and healthcare professionals from the pilot sites to perform interviews assessing barriers and facilitators regarding participation in the trial. Formal qualitative assessments of participants or staff will not take place as part of the research during the main recruitment phase.

## Withdrawal of participation and consent criteria

Participants may withdraw their consent for the trial at any time if they choose not to continue, without prejudice for future care and they do not need to provide a reason why. However, if they are willing to provide a reason, it should be recorded on the relevant CRF. Participants will be asked to what extent they wish to withdraw their consent from the trial:

Prior to trial intervention, i.e., withdrawal from the trial intervention:

The participant would like to withdraw from their randomly allocated trial treatment i.e., they will not undergo the type of surgery as determined by randomisation, but will remain in trial follow-up and data will continue to be collected from them.

Post-trial intervention:

- Participant would like to withdraw from the attendance at clinic visits but gives permission for the trial related data to be collected from medical notes, NHS digital or contacted remotely (e.g., via telephone) to collect trial data
- Participant would like to withdraw from all trial assessments (both in person and remotely) but is happy for the study team to collect data from NHS databases in the future
- Participant would like to withdraw from the trial completely – no further data collection will be carried out

Withdrawal status will be documented on trial CRF and medical records. It is important to note that the data already collected prior to the point of withdrawal can still be analysed and included in the trial dataset.

## Assessment and management of risk

All clinical trials can be considered to involve an element of risk and in accordance to LCTU operating procedures, EVOCC has been assessed for any risks relating uniquely to this trial. Both surgical interventions are routinely used to treat this patient population therefore it has been concluded that the trial carries no higher risk than that of standard medical care.

## Pandemic adaptations

In the event of a pandemic (e.g., COVID-19) or local/national lockdowns which may affect EVOCC participants, sites should check ahead of scheduled trial visits that participants are able to attend in line with local Trust policy. Participants should follow government guidelines with regards to testing and self-isolation and MUST inform their Research Nurse/trial team if they are experiencing symptoms, have tested positive and/or must self-isolate. All scheduled trial visits can be rescheduled and certain data/assessments can be collected/performed remotely e.g. over the telephone or through medical notes.

Participant safety data will be collected during the course of the trial as usual.

In some cases, the data collection may be limited to primary endpoints, i.e., deaths and amputation data.

## End of trial

The end of the trial will occur when the final data capture takes place.

# SAFETY REPORTING

## Definitions

| **Term** | **Definition** |
| --- | --- |
| **Adverse Event (AE)** | Any untoward medical occurrence in a patient or clinical investigation participants, which does not necessarily have to have a causal relationship with this treatment.  An AE can therefore be any unfavourable and unintended sign (including an abnormal laboratory finding), symptom or disease temporally associated with the study, whether or not considered related to the study. |
| **Adverse Reaction (AR)** | An untoward and unintended response in a participant to an investigational medicinal product which is related to any dose administered to that participant.  The phrase "response to an investigational medicinal product" means that a causal relationship between a trial medication and an AE is at least a reasonable possibility, i.e. the relationship cannot be ruled out.  All cases judged by either the reporting medically qualified professional or the Sponsor as having a reasonable suspected causal relationship to the trial medication qualify as adverse reactions. It is important to note that this is entirely separate to the known side effects listed in the SmPC. It is specifically a temporal relationship between taking the drug, the half-life, and the time of the event or any valid alternative aetiology that would explain the event. |
| **Serious Adverse Event (SAE)** | A serious adverse event is any untoward medical occurrence that:   - results in death - is life-threatening - requires inpatient hospitalisation or prolongation of existing hospitalisation - results in persistent or significant disability/incapacity - consists of a congenital anomaly or birth defect   Other ‘important medical events’ may also be considered serious if they jeopardise the participant or require an intervention to prevent one of the above consequences.  NOTE: The term "life-threatening" in the definition of "serious" refers to an event in which the participant was at risk of death at the time of the event; it does not refer to an event which hypothetically might have caused death if it were more severe. |
| **Expected SERIOUS Adverse Events** | The following events are considered as expected SAEs. For the purposes of this trial such events do **NOT** require reporting to the EVOCC central team or Sponsor. These events should continue to be recorded in the medical records according to local practice and on the routine follow-up CRFs.   \| Bowel obstruction \| \| --- \| \| Acute kidney injury \| \| Arterial dissection \| \| Arterial pseudoaneurysm formation \| \| Atrial fibrillation (new) \| \| Bowel ischaemia \| \| Chest infection \| \| Diabetes (new diagnosis) \| \| Diabetic foot ulcer (new) \| \| Diabetic ketoacidosis \| \| Endovascular re-intervention (will have to be reported separately using the relevant form) \| \| Failure of endovascular closure device \| \| Fall \| \| Fasciotomy \| \| Haematoma (at site of surgery or percutaneous puncture) \| \| Heart failure (non-fatal) \| \| Hernia \| \| Hypertension (new diagnosis) \| \| Ileus \| \| Intermittent claudication (new diagnosis) \| \| Laparotomy \| \| Lower limb compartment syndrome \| \| Lower limb oedema \| \| Minor amputation (below the ankle joint) \| \| Myocardial infarction (non-fatal) \| \| Need for open surgical access to an artery during the index endovascular procedure \| \| Peripheral embolisation \| \| Peripheral embolisation, not requiring surgery or re-intervention \| \| Prescription of antibiotics for lower limb tissue loss (ulcer/gangrene) \| \| Re-admission to hospital within 30 days of randomisation \| \| Requirement for catheterisation (urinary catheter) \| \| Stent or graft occlusion \| \| Stent or graft thrombosis \| \| Stoma formation \| \| Stroke \| \| Surgical re-intervention (will have to be reported separately using the relevant form) \| \| Transient ischaemic attack \| \| Transient renal replacement therapy \| \| Urinary retention \| \| Urine infection \| \| Use of additional stent or additional endovascular device \| \| Venous thrombosis (deep and/or superficial) \| \| Viral infection \| \| Wound infection \| |

## Reporting procedures for All serious Adverse Events

### Reporting procedures for Adverse Events

AEs will not be reported as part of this trial. AEs should be recorded in the medical records according to local practice.

### Reporting Procedures for Serious Adverse Events

Adverse events which meet the serious criteria (SAEs) and which are deemed related to the trial, except those identified as expected within section 9.1, occurring from point of randomisation and until follow-up completion (minimum of two years after trial treatment), must be reported to the Sponsor and the EVOCC trial management team immediately and within 24 hours of becoming aware of the event. The SAE will be reported using the current standard Sponsor SAE reporting form and according to the Sponsor SOP for reporting serious adverse events. For clarity, those listed in the above table as expected SAEs and SAEs that are deemed as **not** related to the trial do not need to be reported, only those events that fall outside of this list should be reported expediently as described. All exempt SAEs should be recorded in the patient medical notes as such.

**Please note:** **No serious adverse reactions (SARs) are anticipated as a unique consequence of participation in the EVOCC trial.**

The following information will be recorded for all SAEs:

- Description of the event
- Date of onset and end date
- Severity
- Assessment of relatedness to trial
- Action taken.

SAEs will be followed until resolution or the event is considered stable.

It will be left to the investigator’s clinical judgment whether or not an SAE is of sufficient severity to require the participant’s removal from the trial. The participant must undergo an end of trial assessment and be given appropriate care under medical supervision until symptoms cease or the condition becomes stable. The severity of events will be assessed on the following scale: 1 = mild, 2 = moderate, 3 = severe. The relationship of SAEs to the trial will be assessed by a medically qualified investigator.

Additional information will be provided if requested to the Sponsor and main Research Ethics Committee (REC). The Principal Investigator or another delegated physician (as agreed by the Sponsor) is responsible for the review and sign off of the SAE and the assessment of causality.

The Sponsor will perform an initial check of the information and ensure that the SAE line listing is reviewed by the Director of Research & Innovation. All SAE information must be recorded on an SAE form and sent to the Sponsor. Additional information received for a case (follow-up or corrections to the original case) needs to be detailed on a new SAE form and sent to the Sponsor.

Copies of all documentation and correspondence relating to SAEs will be stored in the TMF and ISF.

In addition to the expedited reporting above, the CI shall submit once a year throughout the trial or on request an Annual Report to the Ethics Committee which lists all SAEs that have occurred during the preceding 12 months.

The trends in these events will be independently reviewed in addition to the trial safety monitoring procedures. The decision regarding the frequency of review of individual and cumulative SAEs is to be based on the trial design, risk assessment and advice from the Sponsor / TSC / DMC and will include:

- Clinical review of a line listing of all life threatening or SAEs resulting in death within 1 week of their occurrence
- Clinical review of a line listing of all other SAEs on a monthly basis
- Cumulative review of all safety information by the DMC on an annual basis or more often if required

### Reporting Urgent Safety Measures

The Sponsor, the Chief Investigator or the local Principal Investigator at a research site may take appropriate urgent safety measures in order to protect research participants against any immediate hazard to their health or safety.

If any urgent safety measures are taken the CI/Sponsor shall be notified immediately, and in any event no later than 3 days from the date the measures are taken, give written notice to the Sponsor and the relevant REC of: the measures taken; reason these measures taken; the circumstances giving rise to those measures and the plan for further actions.

# ROLES AND RESPONSIBILITIES OF TRIAL MANAGEMENT COMMITEES/GROUPS & INDIVIDUALS

## Trial Steering Committee (TSC)

The role of the TSC is to provide overall supervision for the trial on behalf of the Sponsor and Funder and to ensure that the trial is conducted to the rigorous standards set out in the Department of Health’s UK Policy Framework for Health and Social Care Research and the Guidelines for Good Clinical Practice. The remit of the TSC is to provide advice, focus on trial progress and adherence to study protocol, patient rights, safety and well-being as well as considering new information and its relevance to the research question. The TSC will also ensure that all ethical and regulatory approvals are obtained and agree proposals for substantial protocol amendments.

The TSC will be composed of an Independent Chair, and at least two Independent Clinicians or others with expertise relevant to the trial, along with a lay/PPIE representative. NIHR and Sponsor Representatives may be invited to attend TSC meetings as observers if required. The TSC will adopt a charter to define its terms of reference and operations. The TSC will have a majority independent representation, including the Chair, meet on an annual basis and send reports and make recommendations to the TMG/Sponsor/Funder. Members will be required to sign a ‘conflict of interests declaration form’.

## Data Monitoring Committee (DMC)

Independence is a key characteristic of a Data Monitoring Committee where the committee members are completely uninvolved in the running of the trial and thus cannot be unfairly influenced (either directly or indirectly) by individuals or institutions, involved in the trial. The role of the DMC is to monitor outcome data, safety data and other trial data and make recommendations to the TSC on whether there are any ethical or safety reasons why the trial should not continue. The safety, rights and well-being of the trial participants are of paramount importance, in addition to the validity of the trial data. The DMC will also consider data emerging from other related trials, provided by the Sponsor or Funder.

The DMC will consist of an Independent Chair, an Independent Clinician and an Independent Statistician. The DMC will adopt a charter, based on the principle of Damocles, to define its terms of reference and operation. The DMC will meet on annually as specified in the charter or more regularly in response to safety concerns or on recommendations by the committee themselves.

## Trial Management group

The TMG will be responsible for the day-to-day management of the trial to ensure the delivery through delegation of tasks to individuals or a group, for all aspects of the trial, including recruitment rate, budget management, safety reporting, protocol compliance and ensuring appropriate action is taken to safeguard trial participants and quality of the trial. The TMG members will meet regularly to ensure all practical details of the trial are progressing and working well.

# STATISTICS AND DATA ANALYSIS

## Sample size calculation

Calculations are based on our published meta-analysis, cohort study across multiple NHS hospitals treating patients with symptomatic PAD and aorto-iliac disease TASC II C and D with endovascular means, and contemporary NHS event rates in our HES analysis specifically for this trial (unpublished data). The perioperative mortality rate (at 30 days) for endovascular treatment is 1% in this unadjusted observational (non-randomised) data and 3% for surgery. Overall, 43% of patients undergoing endovascular treatment for these indications are expected to require re-intervention within 3 years. Based on a 20.63% event rate per year for endovascular treatment regarding amputation and/or death (national HES data), the proportion of patients for this arm free from death and amputation at 3 years is expected to be 50%. Further, based on HES data, the effect of both open surgery and endovascular treatment on the primary outcome is non-linear (more events in the first few months after the intervention), therefore the power calculation is based on an analysis with restricted mean survival time(8). Assuming a 2 year recruitment period and 3 year median follow-up, a sample size of 596 patients (accruing 256 events during follow-up) will provide 90% power (2 sided a=0.05) to detect an absolute risk reduction of 13.3% in terms of event-free survival (death or amputation) at 3 years (50% vs. 63.3% with a hazard ratio of 0.66) between arms (open surgery vs. endovascular treatment). This equates to an increase in restricted mean survival time (for death and/or amputation) of 3 months. Allowing for a conservative 5% loss of follow-up data (based on the completed NIHR HTA BASIL trials and given that the composite comprises of death or major amputation), the final sample size is 628 i.e. 314 participants per trial arm. Patients will be minimised based on age (above 65 years), sex and presence of tissue loss (yes/no) and stratified by site, which are the main factors predicting outcome after treatment for severe symptomatic PAD in multiple studies and similar NIHR HTA trials.

## Planned recruitment rate

The progression criteria for the internal pilot are based on 10 sites each recruiting on average 1 participant per month for 6 months. This equates to approximately 6% of eligible patients consenting to the trial. If the progression criteria from the internal pilot are met, we aim to recruit during the main phase from over 30 NHS sites over a further 2 years. Based on HES, a total of 2,200 eligible open surgery and 4,000 endovascular treatment procedures take place per year across approximately 30 of the vascular hospital networks that will take part in EVOCC. The targeted recruitment rate will remain at 6% throughout the main trial recruitment period.

## Statistical analysis plan

The Leicester Clinical Trials Unit (LCTU) statistician will be responsible for planning, monitoring and carrying out data analyses throughout the study, supervised by the senior LCTU statistician (S Barber). Statistical analysis will be undertaken according to a pre-specified statistical analysis plan written prior to database lock and reported following CONSORT statements (20).

## Summary of baseline data

Data collected at baseline will be presented as means and standard deviations or medians and ranges if not normally distributed for all continuous variables and/or number and percentage for categorical variables, overall and by randomised group. No formal statistical hypothesis tests will be used to assess normality assumptions or make comparisons of differences between groups.

## Primary outcome analysis

The primary analysis of the primary outcome (amputation free survival) will compare the time to first event between randomised groups. The primary outcome will be defined as time to first event calculated by measuring in days the time from randomisation until first event or last known event-free observation during the follow-up period. Participants not experiencing a primary outcome event will have their time to event censored at last follow-up date. Participants withdrawing consent or that are lost to follow-up prior to reaching primary outcome will have their time to event at last contact date. The primary analysis will be undertaken on an intention-to-treat basis with all participants included post randomisation analysed in the group to which they were allocated. The primary outcome will be compared between randomised arms using restricted mean survival time carried out using a validated programme such as the Stata module ‘STRMST2’ or equivalent. A point estimate, 95% confidence interval and 2 sided p-value will be presented. Statistical significance will occur if the p-value is <0.05. The primary analysis will be adjusted for the minimisation factors used at randomisation. The individual components of the primary outcome will be reported separately as secondary outcomes

Secondary analyses of the primary outcome will explore a per-protocol population excluding major protocol deviators.

## Secondary outcome analysis

Secondary outcomes will be analysed on a complete case population using appropriate methods: restricted mean survival time or cox proportional hazards regression (as appropriate) for time-to-event outcomes, logistic regression for binary outcomes, linear regression for normally distributed continuous outcomes or where not normally distributed using appropriate non-parametric methods and mixed effects models with participant as random effect where outcomes are repeated measures over time, all adjusted as described and presented as point estimates with 95% confidence intervals in the intention to treat population.

### Subgroup analyses

An un-powered exploratory subgroup analyses of the primary outcome will be performed to assess associations between TASC class of aorto-iliac disease, length and location of aortic and iliac occlusions, and types of endovascular treatment (e.g. use of kissing stents aorto-iliac stents, use of covered vs. uncovered stents, and use vs. no use of an aortic stent), based on our published evidence and feedback from NHS survey which strongly supported this exploratory analysis (8, 9).

### Interim analysis and criteria for the premature termination of the trial

There will be no formal interim statistical assessment for early termination of the trial for safety or efficacy. The DMC will monitor accruing trial data for quality and safety concerns and present their recommendations to the TSC. There are no formal stopping rules associated with DMC reporting.

Data collected during the 6 month internal pilot will be presented descriptively in terms of the progression criteria to the oversight committees and funding body and used to assess if it is feasible to continue to recruit to the main study.

### ADVERSE EVENTS REPORTING

Unexpected serious adverse events (SAEs) outside of the list provided in section 9.1, will be reported descriptively in the safety population i.e., participants undergoing trial treatment, presented in groups according to treatment received not as randomised. The number of unexpected serious adverse events and the number of participants experiencing unexpected serious adverse events will be presented categorised by common SAE terms. Unexpected serious adverse events will be presented as line listings and grouped by severity.

### Procedure(s) to account for missing or spurious data

Missing data is expected to be minimal given the type and mode of data collected. The primary outcome other time-to-event outcomes will not encounter missing data due the nature of the data. Furthermore, any losses to follow-up should be minimised by the intended methods to capture long term follow-up through routine data capture.

### Economic evaluation

A prospectively planned economic evaluation will be conducted from an NHS and personal social services perspective, as per National Institute of Care Excellence (NICE) recommendations (21), led by Health Economists with considerable experience in NIHR HTA funded vascular research. Use of hospital and community services will be recorded over the trial time horizon using the trial CRFs. Quality-of-life will be assessed using the EQ-5D-5L, converted to health utility scores using the UK value set recommended by NICE. Unit costs will be based on manufacturers’ prices and national databases. Days lost from work and activities will be recorded and used in a secondary analysis. The extent and pattern of missing data will be assessed and appropriate methods employed, for example, multiple imputation.

If the trial finds a clinically meaningful difference in outcomes or costs over the study time horizon, a decision model will be constructed to take account of the loss in life years and long-term impact on quality-of-life after complications. A state-transition decision model (Markov model) will estimate costs and QALYs over the lifetime of the cohort. The recommended discount rate of 3.5% per year will be used. Parametric survival curves will be used to estimate rates of death, cardiovascular disease and amputation events. Long term rates of events will be estimated based on extrapolation of study data and literature. Outcomes or states of the model will be decided based on consultation with experts and review of previous economic evaluations in PAD. Tunnel states may be used if time to death following non-fatal events is time-varying. Sensitivity analyses will be conducted by varying key variables with high uncertainty. The model will be constructed, analysed, and reported, according to CHEERS guidelines (22).

# DATA MANAGEMENT

## Data flow diagram

Please refer to **Appendix 2** for the trial Data Flow Diagram.

## Data handling and record keeping

All data handling and record keeping will be kept in adherence to University of Leicester’s and NHS Organisation(s) policies.

### National Data Opt-out

The national data opt-out was introduced on 25th May 2018 enabling patients to opt out from the use of their data for research or planning purposes in line with the recommendations of the National Data Guardian in their review of Data Security, Consent and Opt-outs. As from 1st August 2022, all NHS Trusts are now compliant with the national data opt-out operational policy guidance document; for further information please visit <https://digital.nhs.uk/services/national-data-opt-out/operational-policy-guidance-document>.

### Data Collection Tools and Source Document Identification

LCTU will be responsible for Data Management for the trial and will undertake data validation, database queries/reviews in line with their SOPs.

ICH E6 section 1.51, defines source data as "All information in original records and certified copies of original records or clinical findings, observations, or other activities in a clinical trial necessary for the reconstruction and evaluation of the trial. Source data are contained in source documents (original records or certified copies)."

The basic concept of source data is that it permits not only reporting and analysis but also verification at various steps in the process for the purposes of confirmation, quality control, audit or inspection. A number of attributes are considered of universal importance to source data and the records that hold those data. These include that the data and records are:

- Accurate
- Legible
- Contemporaneous
- Original
- Attributable
- Complete
- Consistent
- Enduring
- Available when needed

Source Data is defined as the first place where data is recorded, this will include:

- Medical Records
- Paper CRFs
- Participant reported outcome questionnaires
- Laboratory reports
- Printouts from equipment
- Field notes
- Audio-recordings

Data collection tools will comprise of:

- Macro Database (transcribed from CRFs) and direct source data entry
- Participant reported outcome questionnaires

Audio recordings of clinical consultations taken as part of the research undertaken by the QuinteT team will be captured on encrypted audio-recording devices, which the QuinteT team will provide to sites (with instructions). Recordings will be periodically transferred securely to the University of Bristol research team using a Trust-approved secure data transfer system (e.g. BOLT). The recordings will be transcribed and de-identified by the University of Bristol or a University of Bristol approved contracted transcribing service that has signed the University of Bristol’s Confidentiality Agreements. Transcripts and recordings will be held up to 6 years from the end of the trial on a secure database at the University of Bristol which will only be accessed by authorised members of staff in the QuinteT team. For transcripts only, we will request separate permission for these to be stored indefinitely on a separate data repository (so other QuinteT researchers at the University of Bristol can apply for permission to access the transcripts for future ethically approved research). Any paper copies of the transcripts will be stored securely in a locked filing cabinet at the University of Bristol and destroyed 6 years after the end of the trial.

Digital recordings taken as part of the process evaluation will be stored on a secure University of Leicester server and will be deleted following transcription and anonymisation of the data. Recordings will be accessible only to essential personnel. All quotations or descriptions used in reports will be anonymised, and details may be altered to further protect anonymity. Field notes from observations will also be anonymised.

Both research teams will provide information on data storage to the central co-ordinating team at LCTU.

### Data Handling and Record Keeping

Records of trial participant data will be made on trial specific electronic CRFs. Trained member(s) of the site research team will enter data directly into a commercially available web based Clinical Data Management System (CDMS) provided by the LCTU, called MACRO. On-entry validation checks will be applied where required and data entered will be checked for completeness, accuracy and timeliness by the site research team/trial manager, with queries managed using the data clarification functionality within the CDMS system.

A copy of the participant Informed Consent Form and Information Sheet will be given to the participant, a copy will be placed in the hospital notes of all participants and original copies in the Investigator Site File. A sticker will be placed on the cover of the notes (or inside cover) detailing the trial title, contact details of the PI and the fact that the notes should not be destroyed for 6 years from the end of the trial. All trial visits and related AEs/SAEs will be recorded in the hospital notes. Where electronic or hybrid medical notes are used it is expected that electronic flags, scanned documents and annotation are included in the medical notes.

All data collected will be stored in securely locked filing cabinets and in password-protected databases. After the trial is complete, all data will be securely archived and will be destroyed after six years. Anonymised data will be stored in secure specialist data centre/repository relevant to this subject area and available for future research, should the participant consent to data storage.

During the trial, any paper CRFs and source data documentation will be stored in a secure area accessible to trial site staff. Each enrolled participant will be allocated a unique study ID so that the CRFs and electronic database remains pseudonymised.

According to the ICH guidelines for Good Clinical Practice, the trial management team may check the CRF entries against the source documents, except for the pre-identified source data directly recorded in the CRF. LCTU will develop a monitoring plan for source data verification (SDV) checks. The informed consent form will include a statement by which the participant allows the Sponsor and LCTU’s duly authorised personnel, the Ethics Committee, and the regulatory authorities to have direct access to original medical records which support the data on the CRFs (e.g., participant’s medical file, appointment books, original laboratory records, etc.) in the event that this trial is monitored by the trial Sponsor. These personnel must maintain the confidentiality of all personal identity or personal medical information (according to confidentiality and personal data protection rules).

A Data Management Plan will be created with specific details on data handling and record keeping.

## Access to Data

Direct access will be granted to authorised representatives from the Sponsor, host institution, LCTU, and the regulatory authorities to permit trial-related monitoring, audits and inspections in line with participant consent.

All trial documentation will be retained in a secure location during the conduct of the trial. Personal identifiable data will be retained by each participating site for a maximum of 12 months following the end of the trial, after which it will be destroyed, unless participants have expressed an interest in being invited to the results dissemination event and/or receiving a copy of the trial newsletter. In these circumstances, personal identifiable details such as names and contact details will be retained on a password protected database until required, and then destroyed. The University of Leicester will also hold identifiable data for potential future data linkage, to assess future outcomes (amputation or mortality) on The University Research Filestore Service (RFS) secure environment.

All electronic data will be stored on secure network systems, to which only the relevant trial personnel will have access.

For the purposes of this trial, the University of Leicester will act as the Data Controller for data held on the Clinical Data Management System (CDMS). The University of Bristol will act as the Data Controller for data generated as part of the qualitative analysis. The University of Leicester will act as the data controller for the data held on the NVivo qualitative data indexing software as part of the process evaluation. NVivo transcription data is encrypted both in transit and at rest and only the account owner has access to and control over the data.

## Archiving

Personal identifiable data generated by the trial will be retained for 6 years following the notification of the end of the trial before being destroyed in a confidential manner.

Following completion of the trial data analysis, data and essential trial records, including the final trial report, will be archived in a secure location for at least 6 years after the completion of the trial, in accordance with LCTU SOPs. No trial-related records, including hospital medical notes, will be destroyed unless or until the Sponsor gives authorisation to do so.

Details of the University of Leicester archiving Standard Operating Procedure can be found at: <http://www2.le.ac.uk/offices/ias>.

# MONITORING, AUDIT & INSPECTION

The University of Leicester, as Sponsor, operates a risk-based monitoring and audit programme, to which this trial will be subject. The LCTU operates a risk-based Quality Management System, which will apply to this trial with quality checks and quality assurance audits performed as required.

As part of the quality management process, the trial will be subject to a risk assessment which will be developed by the LCTU in association with the Sponsor. The trial manager will undertake quality checks and assurance audits to ensure compliance with protocol, ICH GCP, and regulatory requirements.

All source data, trial documents, and participant notes will be made available for monitoring, audits and inspections by the Sponsor (or their delegate), NHS Host Organisation and the regulatory authorities, should a monitoring visit be undertaken.

# ETHICAL AND REGULATORY CONSIDERATIONS

## Research Ethics Committee (REC) review AND reports

Once the initial Sponsor review process is complete and a Sponsor reference number has been allocated and all requested documentation has been received and checked, authorisation from the University of Leicester’s Research Governance Office will be issued to book further reviews of the proposed research. The NHS Research Ethics Committee and the Health Research Authority will then review the proposal. Agreement in principle is subject to the research receiving all relevant regulatory permissions. Submission for regulatory approvals will be submitted via Integrated Research Application System (IRAS). The Chief Investigator will ensure that all regulatory approvals, confirmation of capacity and capability from NHS sites and Sponsor greenlight are in place before participants are approached.

For any required amendment to the trial, the Chief Investigator, in agreement with the Sponsor will submit information to the appropriate body in order for them to issue approval for the amendment. Amendments will be implemented upon receiving Sponsor Green Light. The Research Governance Office’s Standard operational procedures will be followed for the duration of the trial. Amendments will be submitted to the Sponsor in the first instance for review and approval.

Annual progress reports will be submitted to the Ethics Committee annually on the anniversary date of when favourable opinion was given to the Chief Investigator.

The Chief Investigator will notify the REC when the trial has ended by completing the end of trial notification form and will submit a final report of the results within one year after notifying the REC.

A trial master file will be maintained for the duration of the trial and will be stored for 6 years after the trial has ended.

## Peer review

The trial design and protocol have undergone a high-quality peer-review both internally (University of Leicester) and externally by six lay individuals and eight experts, as well as the NIHR HTA panel which funded this research.

## Public and Patient Involvement (PPI)

**PPI to support trial design**

Three PPI focus groups were convened to develop this trial, led by the chief applicant and PPI support staff at the NIHR Leicester Biomedical Research Centre. 11 patients who had endovascular (keyhole) artery treatment, 7 who had open surgery, 3 full-time carers and 4 partners took part, from multiple ethnic and socioeconomic backgrounds. Most patients had diabetes with 5 having previously had a leg amputation following arterial intervention. We explored outcomes of interest, follow-up procedures, study acceptability, and clinically important differences with all participants. All agreed that the main outcome measure should include mortality and amputations (equally weighted), which are the two things that matter most to patients as well as their partners and carers. They specifically supported that follow-up should continue for a minimum of 2 years; patients reported that they keep needing re-interventions several months after their initial intervention, especially when they have endovascular treatment, and this might also lead to amputation. All patients expressed that they would take part in the trial and found randomisation an acceptable concept. In fact, they found that the uncertainty of not knowing which procedure is best in terms of saving their life or leg is a major stress factor for them and they would like to see a trial address this important question. When asked, they strongly supported a superiority design, which is reflected in our current design. These patients reviewed and approved the trial design including outcomes of interest, duration, types of quality of life assessments, and they also approved the relevant plain English summary. Most importantly, the patients in our focus group led our choice of primary outcome and the duration of the primary outcome assessment and clinically important difference. They unanimously found that they suffered many complications and amputations after these interventions. Not losing their leg, not dying and keeping as much of their autonomy as they can are the most important things for them. This is why even a modest difference of 3 months extra survival or keeping their leg after intervention was so important for them and has therefore been chosen as the primary outcome on which EVOCC is powered. Overall, every element of EVOCC is grounded on patients’ and families’ views.

Besides our patient partners, to explore equipoise amongst healthcare staff nationally, we performed an online survey of 60 surgeons, 23 radiologists, and 8 nurses. This covered 38 NHS sites (64% of vascular centres in the NHS) and was supported by various vascular professional networks in the NHS, including representation from all professions involved in vascular care. Overall, 97% of the participants agreed this trial is urgently needed; 88% felt it would be possible to deliver within a 2 year recruitment period. Finally, 91% of surgeons and radiologists felt they were in equipoise and would recruit/randomise patients in this trial. The healthcare staff who took part in this survey have again reviewed and approved our final trial design prior to the Stage 2 application, confirming that it reflects the needs of the NHS and can be delivered as planned within existing NHS pathways of care for peripheral arterial disease nationally.

**PPI during trial delivery**

From our diverse PPI group which supported trial design (25 patients, family members, and carers), 2 formal PPI co-applicants were identified: a female and a male patient (different ethnic backgrounds) who have peripheral arterial disease and have previously undergone open and keyhole surgery procedures in the NHS (at several different hospitals). They have already received training in research methodology, including randomised trials, by the lead applicant and staff at the NIHR Leicester Biomedical Research Centre (BRC). They helped us with trial design and they are already familiar with all elements of the trial. They will join all trial meetings, review study materials and processes, and support dissemination during and upon trial completion. If additional training or support of any form is required, this will be provided to them by the lead applicant and our existing PPI support staff (nurses, co-ordinators, patient liaisons, psychologists, and relevant administrative staff at the NIHR Leicester BRC). They will also lead the final dissemination event which we will host upon completion of the trial to share findings with both patient networks and NHS staff nationally. The core trial team will meet at least 3 monthly with our two PPI co-applicants. The two PPI co-applicants will also liaise directly with the lead applicant and our PPI co-ordinator ad hoc (as necessary), to review trial conduct, documents, address any trial-related issues needing immediate patient input, and to prepare regular lay progress summaries, which we will publish online and send to all relevant vascular NHS networks across specialties.

Our two PPI co-applicants will also in turn liaise with a trial-specific PPI Special Advisory Group (SPAG) which will convene when necessary and at least 6 monthly during the trial. We have already identified 8 patients and family members for our PPI SPAG from a wide variety of ethnic and social backgrounds. The SPAG will ensure that the trial truly represents the views of people with severe peripheral arterial disease treated in the NHS, as this is one of the most challenging groups of patients in terms of both socioeconomic background and co-morbidities.

All PPI activities will be supported by an experienced independent co-ordinator co-located with the lead applicant. We will host PPI events either face-to-face or online, based on the specific needs of each event, our patient partners, and issues which might relate to COVID or other travel restrictions. We will use existing PPI infrastructure in the NIHR Leicester BRC (including both staff and equipment). The lead applicant, with considerable PPI experience in several ongoing NIHR vascular studies and our PPI co-ordinator will co-lead all these activities.

Travel and online conferencing support will be available to PPI partners at all times. Further, our BRC will provide translation and language support if that is necessary at any point, to encourage the participation of minorities and ethnic groups in all PPI events, given the high prevalence of the disease in those populations.

## Regulatory Compliance

Before the start of the trial, approval will be sought from a REC for the trial protocol, informed consent forms and other relevant documents. Any substantial amendments that require review by REC will not be implemented until the REC grants a favourable opinion for the trial.

All correspondence with the REC will be retained in the Trial Master File and an annual progress report (APR) will be submitted to the REC by or on behalf of the CI within 30 days of the anniversary date on which the favourable opinion was given and annually until the study is declared ended.

The Chief Investigator will notify the REC when the study has ended by completing the end of study notification form and will submit a final report of the results within one year after notifying REC.

## Protocol compliance

Prospective, planned deviations or waivers to the protocol are **not** permitted under the UK regulations on Clinical Trials and must not be used e.g., it is not acceptable to enrol a participant if they do not meet the eligibility criteria or restrictions specified in the trial protocol. However, accidental protocol deviations can happen at any time and they must be adequately documented on the relevant forms and reported to the Chief Investigator immediately.

Deviations from the protocol which are found to frequently recur are not acceptable, will require immediate action and could potentially be classified as a serious breach.

If a protocol breach occurs, then the CI will document this in adherence to the University’s Standard Operational Procedure SOP Identifying and Reporting Deviations and Serious Breaches of GCP and/or the Protocol for Trials. The CI will seek advice from the research supervisors and the Sponsor. All serious breaches must be reported to Sponsor immediately and within 24 hours of identification.

## Data protection and patient confidentiality

All information collected in the study will be kept strictly confidential.

The Chief Investigator will have access to the trial documentation and will be the data custodian.

The investigator will comply with the requirements of the General Data Protection Regulation (and other applicable regulations) with regards to the collection, storage, processing and disclosure of personal information and will uphold the Act’s core principles.

Analysis of the generated data will be undertaken by the Chief Investigator and LCTU team members on University of Leicester premises and the Quintet team at the University of Bristol. All collected data and electronic confidential information will be saved on a secure drive at the University of Leicester and the University of Bristol. Any printed confidential material will be kept in a folder in a locked drawer in a secured room in a secure office environment office at the University of Leicester.

Anonymised research data will be stored for six years after the trial has ended. Long-term storing will comply with the University of Leicester archiving Standard Operating Procedure. Details can be found at: <http://www2.le.ac.uk/offices/ias>.

## Financial

This trial has been awarded a grant from the NIHR and financial support will be available for participating sites for trial related research costs.

Local CRN support should be available to support the entry of participants into this trial including PI consent.

PPI representatives who attend scheduled trial visits or focus groups / interviews will be reimbursed with regards to expenses for travel up to £50. Lay individuals who take part in PPI groups will be reimbursed at a maximum of £25 per hour as per NIHR principles.

## Indemnity

Sponsorship and insurance for trial design and management will be provided by the University of Leicester. If a participant is harmed due to negligence, this will be covered by the local NHS Trust(s) indemnity arrangements for all participants in clinical trials. If a trial participant wishes to make a complaint about any aspects of the way they have been treated or approached during the research project, the standard National Health Service complaint system will be available to them. Details of this are made available to participants the PIS.

## Post trial care

Following completion of trial follow-up, participants will resume their regular routine NHS care and follow-up/surveillance as per local clinical pathways.

## Access to the final trial dataset

The Chief Investigator will have access to the full dataset.

Direct access will be granted to authorised representatives from the Sponsor and host institutions for monitoring and/or audit of the trial to ensure compliance with regulations.

# DISSEMMINATION POLICY

Dissemination will take place in lay and expert format, via an annual networking event in the form of a hybrid (online and face-to-face) workshop for both lay audiences and experts from all vascular healthcare backgrounds. This will be a one-day event, led by the EVOCC trial team, NIHR Leicester BRC staff and PPI partners, with support from the PPI co-ordinator and LCTU.

Six monthly lay and expert updates will be shared on a trial website and social media (e.g., Twitter, Facebook), prepared by the PPI partners and EVOCC trial team, including infographics and videos.

We will ensure the widest possible dissemination of both lay and expert outputs to vascular healthcare professionals via the Vascular Society PAD group, which includes members from all disciplines involved in vascular care. Our strategy of publishing outputs in lay and expert format on a trial-specific website, social media and directly communicating these to NHS and vascular healthcare societies, will ensure the widest possible reach during EVOCC and on trial completion. Patient support groups such as the James Lind Alliance (JLA) or Self Help UK will be given lay summaries regularly and will be asked to disseminate amongst their networks. We will ensure that the findings of the trial are immediately disseminated to NICE, since the CI is also a member of a NICE Technology Appraisal Committee. Via our links (co-applicants) to all vascular healthcare societies in the UK, the findings will immediately be disseminated to NHS vascular professionals using mailing/emailing lists.

# references

1. VSGBI. James Lind Alliance Research Priority Setting for Vascular Surgery: JLA; 2021 [cited 2021 11/11/2021]. Available from: <https://www.hyms.ac.uk/research/vascular-psp>.

2. Saratzis A, Jaspers NEM, Gwilym B, Thomas O, Tsui A, Lefroy R, et al. Observational study of the medical management of patients with peripheral artery disease. Br J Surg. 2019;106(9):1168-77.

3. Mohler E, Giri J. Management of peripheral arterial disease patients: comparing the ACC/AHA and TASC-II guidelines. Current Medical Research and Opinion. 2008;24(9):2509-22.

4. Morley RL, Sharma A, Horsch AD, Hinchliffe RJ. Peripheral artery disease. BMJ. 2018;360:j5842.

5. Ambler GK, Thomas-Jones E, Edwards AGK, Twine CP. Prognostic Risk Modelling for Patients Undergoing Major Lower Limb Amputation: An Analysis of the UK National Vascular Registry. Eur J Vasc Endovasc Surg. 2020;59(4):606-13.

6. Salem M, Hosny MS, Francia F, Sallam M, Saratzis A, Saha P, et al. Management of Extensive Aorto-Iliac Disease: A Systematic Review and Meta-Analysis of 9319 Patients. Cardiovascular and interventional radiology. 2021;44(10):1518-35.

7. Saratzis A, Salem M, Sabbagh C, Abisi S, Huasen B, Egun A, et al. Treatment of Aortoiliac Occlusive Disease With the Covered Endovascular Reconstruction of the Aortic Bifurcation (CERAB) Technique: Results of a UK Multicenter Study. J Endovasc Ther. 2021;28(5):737-45.

8. Kloecker DE, Davies MJ, Khunti K, Zaccardi F. Uses and Limitations of the Restricted Mean Survival Time: Illustrative Examples From Cardiovascular Outcomes and Mortality Trials in Type 2 Diabetes. Ann Intern Med. 2020;172(8):541-52.

# Appendices

## APPENDIX 1 - Amendment History

| **Amendment No.** | **Protocol version no.** | **Date issued** | **Author(s) of changes** | **Details of changes made** |
| --- | --- | --- | --- | --- |
| NSA02 | 1.1 | 16-Aug-23 | Carla Richardson, Olivia Howcroft. | Added the ISRCTN Reference and removed the confidentiality agreement on the title page. Updated the Minimisation factors from on age (above 65 years), sex, diabetes (yes/no), chronic kidney disease stage (1 to 4), NHS site, and presence of tissue loss (yes/no) to age (above 65 years), sex and presence of tissue loss (yes/no). Added stratification by site. Updated the data flow diagram in appendix 2. |
| NSA03 | 1.2 | 28-Sep-23 | Olivia Howcroft | Removed the days lost from regular activity assessment from day 30 and removed Details of intervention from day of discharge in table 2.  Updated point 3 in section 6.1.1 to Severe aorto-iliac occlusive PAD defined as “Inter-Society Consensus for the Management of Peripheral Arterial Disease” (TASC II) class C or class D diagnosed on prior cross-sectional imaging.  Updated section 6.3.1 exclusion criteria from Allergy to iodinated intravascular contrast agent including: Iopromide, Iodamide, Iohexol, ioversol, Ioxilan, Iopamidole to Asymptomatic PAD. |
| NSA04 | 1.3 | 29-Sep-23 | Luke Ingram, Carla Richardson | Amended section 6.3.1 to correctly reflect NSA03 change. |
| NSA07 | 1.4 | 17-Oct-23 | Olivia Howcroft | Updated wording in section 8.2 from ‘they will be given sufficient time to make their decision’ to ‘they will be given sufficient time, usually 24 hours, to make their decision.’  Updated section 5 from ‘Patients undergoing these treatments in the NHS are discussed at multidisciplinary (MDT) team meetings to establish equipoise before proceeding with treatment (surgery or endovascular treatment) i.e. the patients will be approached for consent after the MDT discussion has taken place’ to ‘Patients undergoing these treatments in the NHS are usually discussed at multidisciplinary (MDT) team meetings. The patients will be approached for consent once the treating clinicians or MDT feel are in equipoise regarding the treatments.’ |
| SA01 | 1.5 | 13-Jan-2024 | Olivia Howcroft | Updated the wording in section 8.2 to included telephone and written informed consent. Updated the NIHR logo to current logo on title page. Updated the footer for table 2 to ‘Follow up trial visits, with the exception of the 6 months visit, may be undertaken remotely either via telephone or through acquiring information through participant medical records.’ |
| NSA10 | 1.6 | 27-Feb-2024 | Olivia Howcroft, Ana Suazo Di Paola, | Updated the wording in section 5 from ‘The patients will be approached for consent once the treating clinician or MDT feel are in equipoise regarding the treatments’ to ‘The patients will be approached for consent once the MDT feel are in equipoise regarding the treatments’.  Updated key study contacts from Ana Suazo to Ana Suazo Di Paola.  Updated section 11.1 to remove the reference of diabetes, chronic kidney disease stage (1 to 4), NHS site.  Updated the wording in section 6.1.1 from ‘Patient discussed in a local vascular Multi-Disciplinary Team (MDT) meeting and deemed suitable for open surgery or endovascular treatment’ to ‘Patient discussed in any local vascular Multi-Disciplinary Team (MDT) meeting/discussion and deemed suitable for open surgery or endovascular treatment. |
| SA02 | 2.0 | 31-Oct-24 | Anish Patel | Change to the protocol for the addition of electronic consent and for the 6-months follow-up window being increased from +-14 days to +-30 days. These changes have been added to this table (17.1) as v2.1 of the protocol as it was missed in error as part of SA02. |
| SA03 | 3.0 | 30-Jul-25 | Anish Patel | Change in wording to protocol to clarify EVOCC can recruit from more than 30 sites.  Change in wording to explain that if the 6-month follow-up visit cannot be conducted in person then it can be conducted remotely via telephone and obtaining information from medical notes.  Change to section 9, to correct that expected list of SAEs are in section 9.1, not 9.2.1. |

## APPENDIX 2 – data flow diagram


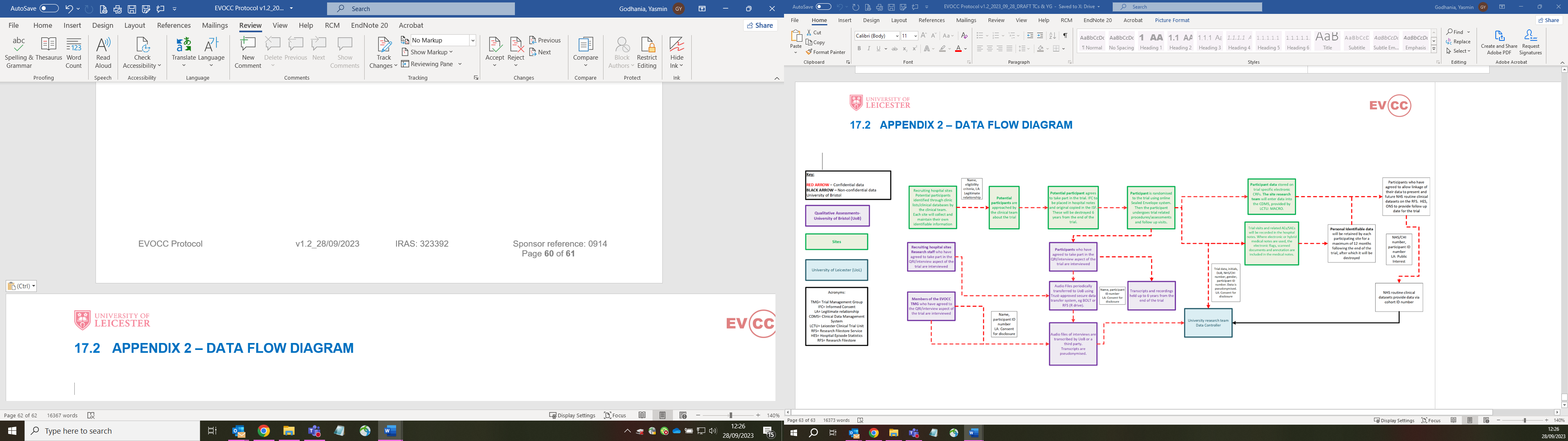

Supplement: online supplemental appendix 1 [file bmjopen-15-10-s001.docx]
