# Supplementary material for: Assessing the clinical and cost-effectiveness of endovascular vs open revascularisation in severe occlusive aorto-iliac disease (EVOCC trial): study protocol for a randomised controlled trial
Source: BMJ Open. 2025 Oct 7;15(10):e106474. doi: 10.1136/bmjopen-2025-106474 (PMC12506155; doi:10.1136/bmjopen-2025-106474)
Supplement: Supplementary file 3 [file bmjopen-2025-106474.reviewer_comments.pdf]

PEER REVIEW HISTORY

BMJ Open publishes all reviews undertaken for accepted manuscripts. Reviewers are asked to complete a checklist review form and are provided with free text boxes to elaborate on their assessment. These free text comments are reproduced below.

ARTICLE DETAILS

Title (Provisional)

A Randomised Controlled Trial Assessing the Clinical and Cost-Effectiveness of Endovascular vs. Open Revascularisation in Severe Occlusive Aorto-Iliac Disease (EVOCC Trial) – Study Protocol for a Trial.

Authors

Saratzis , Athanasios; Davies, Alun; Diamantopoulos, Athanasios; Davies, Robert S.M.; Epstein, David; Jepson, Marcus; Perez, Daniel; Apergi, Despina; Harris, Keith Jonathan; Zayed, Hany; Brookes, Cassandra; Barber, Shaun; Suazo Di Paola, Ana; Ingram, Luke; Richardson, Carla; Patel, Anish

---

This article was not externally reviewed at BMJ Open. Protocols that have been independently assessed prior to submission to BMJ Open are usually fast-tracked to publication on the grounds that further substantial changes will not be possible. This independent assessment will usually be external, independent review for both a substantial grant award from a non-commercial or government funder and ethics approval.
